# Muscle‐directed gene therapy corrects Pompe disease and uncovers species‐specific GAA immunogenicity

**DOI:** 10.15252/emmm.202113968

**Published:** 2021-12-01

**Authors:** Michelle Eggers, Charles H Vannoy, Jianyong Huang, Pravinkumar Purushothaman, Jacqueline Brassard, Carlos Fonck, Hui Meng, Mariah J Prom, Michael W Lawlor, Justine Cunningham, Chanchal Sadhu, Fulvio Mavilio

**Affiliations:** ^1^ Nonclinical, Pharmacology/Toxicology Audentes Therapeutics San Francisco CA USA; ^2^ Jacqueline Brassard Toxicologic Pathology Consulting Tustin CA USA; ^3^ Department of Pathology and Neuroscience Research Center Medical College of Wisconsin Milwaukee WI USA; ^4^ Department of Life Sciences University of Modena and Reggio Emilia Modena Italy; ^5^ Present address: Sana Biotechnology South San Francisco CA USA

**Keywords:** adeno‐associated virus, glycogen accumulation, heart inflammation, lysosomal storage disease, xenogeneic immune response, Genetics, Gene Therapy & Genetic Disease, Musculoskeletal System

## Abstract

Pompe disease is a severe disorder caused by loss of acid α‐glucosidase (GAA), leading to glycogen accumulation in tissues and neuromuscular and cardiac dysfunction. Enzyme replacement therapy is the only available treatment. AT845 is an adeno‐associated viral vector designed to express human GAA specifically in skeletal muscle and heart. Systemic administration of AT845 in *Gaa* ^−/−^ mice led to a dose‐dependent increase in GAA activity, glycogen clearance in muscles and heart, and functional improvement. AT845 was tolerated in cynomolgus macaques at low doses, while high doses caused anti‐GAA immune response, inflammation, and cardiac abnormalities resulting in unscheduled euthanasia of two animals. Conversely, a vector expressing the macaque GAA caused no detectable pathology, indicating that the toxicity observed with AT845 was an anti‐GAA xenogeneic immune response. Western blot analysis showed abnormal processing of human GAA in cynomolgus muscle, adding to the species‐specific effects of enzyme expression. Overall, these studies show that AAV‐mediated GAA delivery to muscle is efficacious in *Gaa* ^−/−^ mice and highlight limitations in predicting the toxicity of AAV vectors encoding human proteins in non‐human species.

## Graphical abstract

Muscle‐restricted expression of GAA via systemic AAV gene therapy clears glycogen accumulation and improves function in a mouse model of Pompe disease. Xenogeneic immune responses in non‐human primates highlight limitations of animal studies to assess toxicity of vectors encoding human proteins.

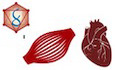

**The paper explained**

**Problem**

The current treatment for Pompe disease, enzyme replacement therapy, is limited by the short half‐life of the enzyme, insufficient uptake by muscle and heart tissues, and immunogenicity. In addition, immunogenicity of the recombinant enzyme represents a formidable challenge in CRIM‐negative patients, for whom therapeutic efficacy is limited even at higher doses and with more intense administration schedules. Gene therapy holds the promise of an efficacious treatment for metabolic diseases such as Pompe disease.

**Results**

We developed a systemic gene therapy, AT845, designed to reconstitute human GAA synthesis and activity directly in skeletal muscle and heart. Systemic administration of AT845 led to a robust, dose‐dependent increase in GAA activity at therapeutic, supraphysiological levels in both mice and non‐human primates, resulting in glycogen clearance and functional improvement in a mouse model of Pompe disease. Toxicity observed at high doses in non‐human primates was due to an anti‐GAA xenogeneic immune response.

**Impact**

Overall, our results show that AT845 leads to a robust, dose‐dependent increase in GAA activity at therapeutic, supraphysiological levels in a Pompe mouse model and non‐human primates, resulting in glycogen clearance and functional improvement in the Pompe mouse model. Lack of immune responses and toxicity in non‐human primates tolerant to the native enzyme supports progression of AT845 to clinical testing. A clinical trial aimed at assessing safety and efficacy of AT845 in patients with CRIM‐positive late‐onset Pompe disease (Clinical trial identifier: NCT04174105) is activated in the United States and is currently recruiting patients.

## Introduction

Glycogen storage disease type II (GSD‐II, OMIM#232300), or Pompe disease (PD), is an autosomal recessive disorder caused by mutations in the lysosomal enzyme acid α‐glucosidase (GAA). GAA deficiency causes lysosomal dysfunction, glycogen accumulation in all tissues, muscle weakness, cardiac and respiratory insufficiency, and neurological abnormalities (van der Ploeg & Reuser, 2008). The clinical manifestations of PD vary from the severe, often lethal infantile‐onset form (IOPD), associated with < 1% residual GAA activity, to the less severe, late‐onset forms (LOPD), characterized by GAA activity up to 20% of normal levels. The only approved treatment for PD is enzyme replacement therapy (ERT), which consists of systemic administration of recombinant human GAA (Kishnani & Beckemeyer, 2014). ERT can extend lifespan in IOPD and slow down or stabilize progression in LOPD (Kishnani *et al*, 2007; van der Ploeg *et al*, 2010; Schoser *et al*, 2017), but its therapeutic efficacy is limited by the short half‐life of the enzyme, insufficient uptake by muscle and heart tissues, and immunogenicity, which elicits a neutralizing antibody response in GAA‐null (CRIM‐negative) patients (Kishnani & Beckemeyer, 2014). In addition, glycogen accumulation impairs lysosomal function, progressively leading to autophagic buildup in muscle fibers that perturbs intracellular trafficking and further reduces exogenous enzyme uptake by receptor‐mediated endocytosis (Fukuda *et al*, 2006a, 2006b).

Gene therapy holds the promise of an efficacious treatment for metabolic diseases (Poletti & Biffi, 2019) and particularly for PD (Colella & Mingozzi, 2019; Salabarria *et al*, 2020). Gene replacement by local or systemic delivery of adeno‐associated viral (AAV) vectors of different serotypes expressing GAA under different promoters showed correction of biochemical and functional parameters in a *Gaa* ^−/−^ knockout (KO) mouse model (Fraites *et al*, 2002; Mah *et al*, 2005; Sun *et al*, 2005, 2008; Elmallah *et al*, 2014; Falk *et al*, 2015). A Phase I/II clinical trial supported the safety of local delivery of an rAAV1‐CMV‐GAA vector to the diaphragm, an approach aimed at ameliorating respiratory insufficiency in affected IOPD patients (Byrne *et al*, 2014; Smith *et al*, 2017). A local delivery approach has also been proposed for skeletal muscle, using an AAV9 vector expressing GAA under the control of the muscle‐restricted desmin promoter (Corti *et al*, 2015). However, local AAV delivery has major drawbacks, such as incomplete correction of the disease manifestations due to poor cross‐correction among muscle fibers, and elicitation of a neutralizing antibody response (Mah *et al*, 2010; Elmallah *et al*, 2014). AAV‐mediated delivery to muscle through a systemic route of administration also raised an anti‐GAA humoral response in GAA‐null mice (Sun *et al*, 2005, 2008; Falk *et al*, 2015; Doerfler *et al*, 2016), although it is unclear whether neutralizing antibodies would affect intra‐fiber GAA synthesis and activity if an antibody response occurred in CRIM‐negative patients (Colella & Mingozzi, 2019). An alternative gene therapy approach to PD is based on systemic delivery of an AAV vector expressing GAA under the control of a liver‐specific promoter (Han *et al*, 2017; Puzzo *et al*, 2017). Liver‐directed synthesis and secretion of GAA could provide a stable source of active enzyme in the circulation, overcoming the pharmacokinetic limitations of ERT, although its availability would remain limited by tissue uptake and may also be affected by neutralizing antibodies.

We developed an AAV8 vector encoding the human GAA protein under the control of a muscle‐restricted promoter/enhancer element (AT845), designed to reconstitute human GAA synthesis and activity directly in skeletal muscle and heart. *Gaa* ^−/−^ mice treated with AT845 by systemic administration showed dose‐dependent reconstitution of GAA expression and activity, glycogen clearance in skeletal and cardiac muscles, and functional improvements as early as 3 months after treatment. GAA activity in murine muscle reached physiological levels at the lowest dose tested (i.e., 3 × 10^13^ vector genomes per kilogram of body weight [vg/kg]). A toxicology study conducted in cynomolgus macaque non‐human primates (NHPs) in the absence of immunosuppression showed anti‐human GAA humoral immune response and elevation of liver enzymes and cardiac biomarkers. Inflammatory infiltrates and functional heart abnormalities were observed at the highest doses tested (> 10^14^ vg/kg). However, animals treated with a vector expressing cynomolgus macaque GAA showed no sign of toxicity nor immune responses at a comparable dose, indicating that the toxicity observed with AT845 was due to an anti‐GAA xenogeneic immune response and not GAA expression *per se*. Overall, our preclinical data show that AT845 is well tolerated when a species‐specific transgene is used and leads to a robust, dose‐dependent increase in GAA activity at therapeutic, supraphysiological levels in both mice and NHPs and to glycogen clearance and functional improvement in GAA‐deficient mice. Lack of immune responses and toxicity in NHPs tolerant of the native enzyme supports the progression of AT845 to clinical testing in CRIM‐positive PD patients.

## Results

### Systemic administration of AT845 shows dose‐dependent correction of GAA expression, enzymatic activity, and glycogen clearance in GAA‐deficient mice

**Figure EV1:**
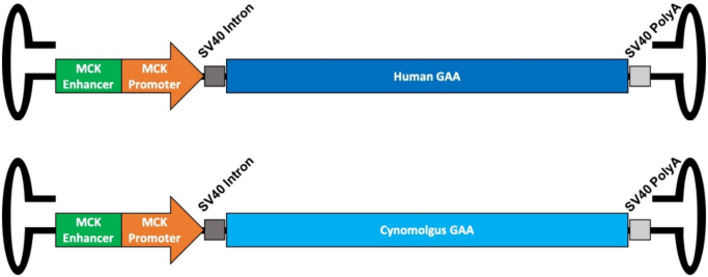
Structure of the rAAV8‐eMCK‐hGAA and rAAV8‐eMCK‐cyno‐GAA vectors Scale is approximate. MCK enhancer: murine MCK enhancer element; MCK promoter: murine MCK promoter; Cynomolgus GAA: cynomolgus acid alpha‐glucosidase cDNA; Human GAA: human acid alpha‐glucosidase cDNA; SV40 PolyA: SV40 late polyadenylation signal sequence.

AT845 (rAAV8‐eMCK‐hGAA) is a serotype‐8 adeno‐associated viral (AAV8) vector that expresses a codon‐optimized cDNA of the human acid alpha‐glucosidase gene (*GAA*) under the control of a murine muscle creatine kinase (MCK) promoter/enhancer combination (Fig EV1). We assessed the *in vivo* efficacy of AT845 by systemic administration in a *Gaa* KO mouse model of PD (B6;129‐*Gaa^tm1Rabn^*/J, hereinafter referred to as *Gaa* ^−/−^). In this study, 72 *Gaa* ^−/−^ mice (nine males + nine females per group) were treated by a single intravenous (IV) injection at 10–11 weeks of age with either vehicle (Ringer's lactate solution) or AT845 at doses of 3 × 10^13^ vg/kg (low dose), 1 × 10^14^ vg/kg (mid‐dose), or 3 × 10^14^ vg/kg (high dose). Mice were followed for 12 weeks after dosing. Additionally, 18 (nine males and nine females) 10‐ to 11‐week‐old wild‐type mice (*Gaa* ^+/+^ littermates) were treated with the vehicle as an additional control cohort.

**Figure 1:**
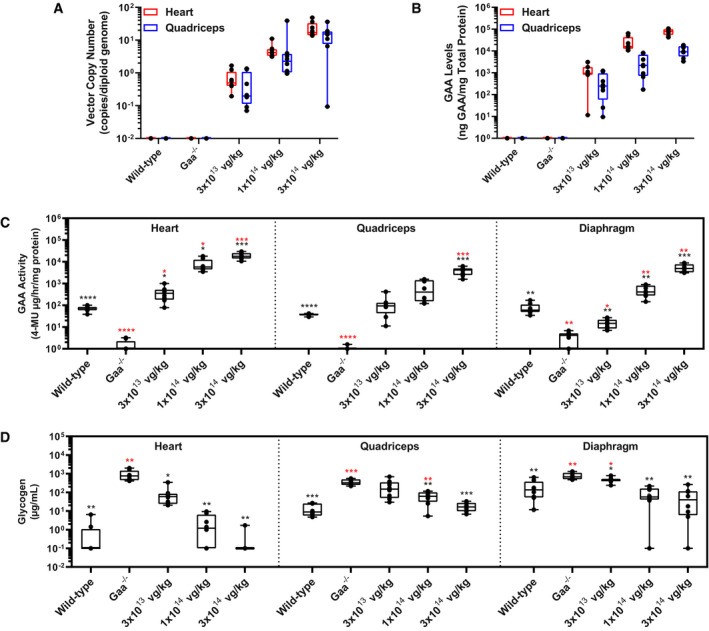
AAV‐mediated gene transfer of an engineered GAA transgene to Gaa^−/−^ mice exhibits dose‐dependent transduction efficiency, increases in GAA activity, and clearance of accumulated glycogen in cardiac and skeletal muscle A–D10‐ to 11‐week‐old mice were treated with vehicle control or AT845 at the vector doses indicated and followed for 12 weeks (*N* = 7 or 8 per cohort). (A) Vector copy number, (B) GAA levels, (C) GAA activity, and (D) glycogen content in cardiac and skeletal muscles. (C, D) Statistical analysis: two‐way ANOVA, Dunnett's test. Data information: Data are presented as box‐and‐whisker plots with Tukey's whiskers that show minimum, median, and maximum values. Asterisks (*) indicate significant differences compared with untreated *Gaa* ^−/−^ mice (black) or wild‐type mice (red). *P < 0.05; **P < 0.01; ***P < 0.001; and ****P < 0.0001. See also Appendix Table S1. Source data are available online for this figure.

To evaluate the transduction efficiency of AT845 in the cardiac and skeletal muscles, we measured AAV vector genome copy numbers (VCN) per diploid genome (vg/dg) by a real‐time PCR test in multiple tissues collected from animals euthanized at 12 weeks post‐treatment. We observed a dose‐dependent increase in VCN in all tested muscle samples with the highest values in the heart (25.2 ± 12.7 vg/dg) and quadriceps (15.1 ± 10.6 vg/dg) at the highest dose (Fig 1A), which translated into a dose‐dependent increase in human GAA protein levels in the same tissues (Fig 1B). GAA enzymatic activity in heart, quadriceps, and diaphragm also increased in a dose‐dependent fashion in treated animals when compared to the same tissues from vehicle‐treated *Gaa* ^−/−^ mice (Fig 1C). GAA activity levels in AT845‐treated *Gaa* ^−/−^ mice exceeded endogenous levels measured in vehicle‐treated wild‐type littermates in the heart and quadriceps at doses ≥ 3 × 10^13^ vg/kg and in the diaphragm at doses ≥ 1 × 10^14^ vg/kg.

Increases in GAA activity correlated with a significant, dose‐dependent reduction in glycogen accumulation in the heart, quadriceps, and diaphragm by 12 weeks after dosing (Fig 1D). The lowest dose of AT845 (3 × 10^13^ vg/kg) resulted in partial but significant glycogen reduction in the heart, diaphragm, and quadriceps muscles isolated from treated mice when compared to vehicle‐treated *Gaa* ^−/−^ mice, while the mid‐dose of 1 × 10^14^ vg/kg resulted in robust reduction of glycogen accumulation in the cardiac and skeletal muscles, with values approaching those of wild‐type littermates (Appendix Table S1). At the highest dose of 3 × 10^14^ vg/kg, AT845 treatment normalized intracellular glycogen levels in the cardiac and skeletal muscles: Most heart samples tested with a minimal residual disease assay resulted in glycogen levels that were below the lower limit of quantitation (LLOQ, Appendix Table S1). Overall, these results indicate that AT845‐driven synthesis of physiological or supraphysiological levels of GAA reverts glycogen accumulation in the muscles of GAA‐deficient mice at doses ≥ 10^14^ vg/kg.

### Administration of AT845 improves skeletal muscle pathology and function in *Gaa* ^−/−^ mice

**Figure 2:**
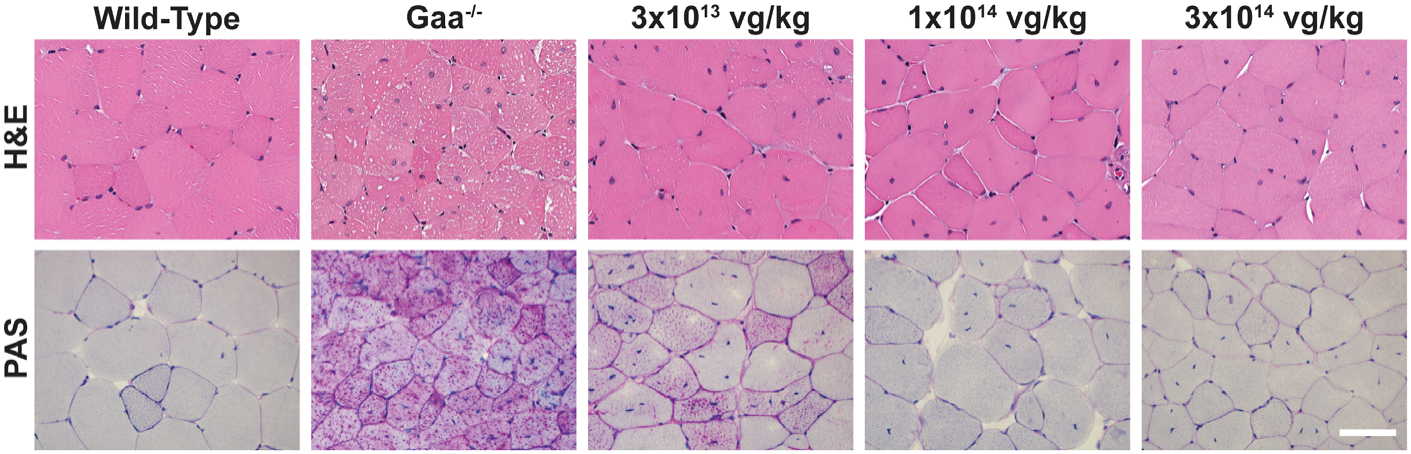
AAV‐mediated transfer of an engineered GAA transgene improves histopathology and exhibits a reduction in glycogen content in *Gaa* ^−/−^ mice Representative images of H&E (top) and PAS (bottom) staining of the quadriceps in wild‐type and *Gaa* ^−/−^ mice untreated or treated with escalating doses of AT845 (3 × 10^13^, 1 × 10^14^, and 3 × 10^14^ vg/kg). Scale bar, 80 µm (see also Appendix Table S1).

On histological examination, quadriceps muscles from vehicle‐treated *Gaa* ^−/−^ mice exhibited minimal‐to‐mild myofiber vacuolation and minimal‐to‐mild degenerative and active regenerative changes after hematoxylin and eosin (H&E) staining (Fig 2, top panels). Periodic acid–Schiff (PAS) staining demonstrated abundant, densely stained pink granules, consistent with aggregated glycogen (Fig 2, bottom panels). AT845‐treated *Gaa* ^−/−^mice showed dose‐dependent reduction in glycogen accumulation in the quadriceps muscles, accompanied by a lack of or marked decrease in the vacuolar, degenerative, and active regenerative changes observed in vehicle‐treated animal muscles (histopathology results are described below). Reduction in glycogen staining was partial at the lower dose and virtually complete at mid‐ and high doses (Fig 2), in accordance with the reduction in glycogen content measured by the biochemical assay (Fig 1D).

**Figure 3:**
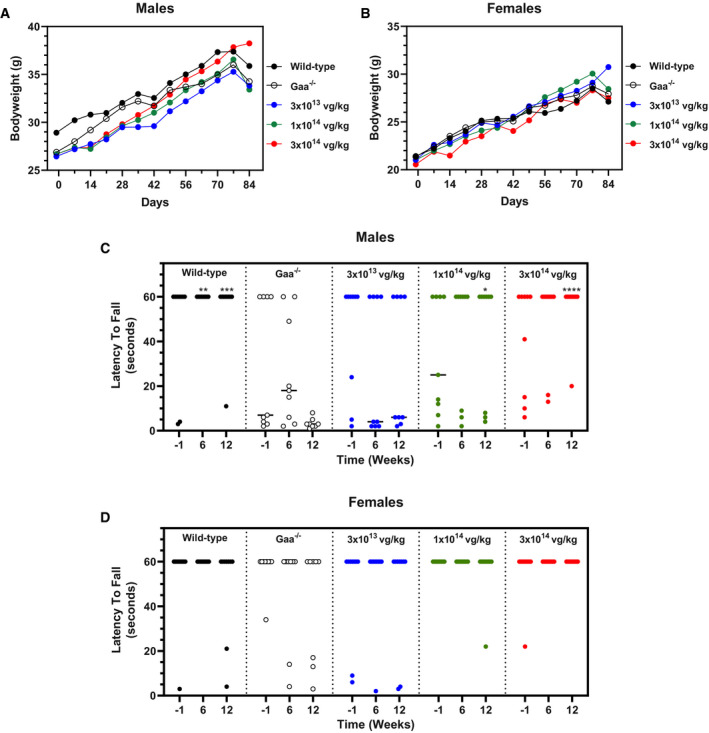
Dose‐dependent functional correction in *Gaa* ^−/−^ mice A–D(A, B) Body weight curves and (C, D) grip response—ability to grip onto an inverted wire screen for a 60‐s period—in wild‐type and *Gaa* ^−/−^ mice untreated or treated with escalating doses of AT845 (3 × 10^13^, 1 × 10^14^, and 3 × 10^14^ vg/kg). All groups had *n* = 9 at all time points, except the 6‐week time point (females) and the 12‐week time point (males and females) for the *Gaa* ^−/−^ mice, which had *n* = 8 per group. Female mice dosed with 3 × 10^13^, 1 × 10^14^, and 3 × 10^14^ vg/kg had a 22, 11, and 0% failure rate at Week 12, respectively; in comparison, the female control WT mice had a 22% failure rate and the control *Gaa* ^−/−^ mice had a 38% failure. Male mice dosed with 3 × 10^13^, 1 × 10^14^, and 3 × 10^14^ vg/kg had a 56, 33, and 11% failure rate at Week 12, respectively; in comparison, the male control WT mice had a 11% failure rate and the control *Gaa* ^−/−^ mice had a 100% failure. Bars represent median. Data information: Data show a dose‐dependent improvement in grip response following treatment with AT845. Statistical analysis: two‐way ANOVA, Dunnett's test. Asterisks (*) indicate significant differences compared with untreated *Gaa* ^−/−^ mice at each respective time point. *P < 0.05; **P < 0.01; ***P < 0.001; and ****P < 0.0001. Source data are available online for this figure.

*Gaa* ^−/−^ mice treated with AT845 at all doses displayed normal weight gain over the time course of the in‐life observation period that was comparable to vehicle‐treated wild‐type littermates (Fig 3A and B). There were no treatment‐related differences in mean body weight values. The treated groups had comparable mean body weight values as the wild‐type and *Gaa* ^−/−^ vehicle control groups, although some fluctuations were noted in all treated male groups that reached statistical significance. However, treated mean values were within 10% of wild‐type controls, and there were no appreciable differences at the end of the 12‐week observation period. It should be noted that vehicle‐treated *Gaa* ^−/−^ mice also showed an almost normal growth curve, which likely reflects the moderate overall pathology of this animal model. To assess muscle function, we tested the ability of each mouse to grip onto an inverted wire screen for a 60‐s period at multiple time points throughout the in‐life portion of the study, including predosing and 6 and 12 weeks after AT845 administration. This test evaluates both isotonic and isometric muscle strength and sensory motor coordination, since it requires mice to reflexively respond to a change in the surface orientation on which they are placed. On average, wild‐type littermates were able to successfully grip onto the inverted screen for the entire 60‐s testing time throughout the observation period, while vehicle‐treated *Gaa* ^−/−^ mice showed a progressive decrease in their grip response that was more apparent in males than in females (Fig 3C and D). By Week 12, *Gaa* ^−/−^ mice failed to grip onto the wire for periods longer than ~3 s. On the contrary, *Gaa* ^−/−^ mice treated with AT845 showed a dose‐dependent improvement in grip response that was apparent as early as 6 weeks after dosing and further improved by Week 12. Low‐dose‐ and mid‐dose‐treated *Gaa* ^−/−^ mice showed partial recovery, resulting in ~50% and ~75% of the mice, respectively, improving their grip response. Interestingly, the response was more robust in female than in male animals at low and mid‐doses (Fig 3C and D), while both male and female mice treated at the high dose showed complete recovery in grip response and were almost indistinguishable from wild‐type littermates at Week 12 (Fig 3C and D). Additional analyses of VCN, GAA, and glycogen data did not provide an explanation for the better performance of females in grip response at the low and mid‐dose. Together, these data demonstrate that administration of AT845 leads to a dose‐dependent reduction in glycogen accumulation in skeletal muscle, normalization of histological parameters, and preservation of muscle strength in *Gaa* ^−/−^ mice.

### Mouse histopathology

Intravenous injection of AT845 to Pompe mice at doses of 0 (vehicle), 3 × 10^13^, 1 × 10^14^, or 3 × 10^14^ vg/kg resulted in test article‐related microscopic findings in the heart, skeletal muscles, and liver. Two *Gaa* ^−/−^ mice in the vehicle group died during the study. In the remaining vehicle‐treated GAA^−/−^ mice (eight males and eight females), H&E‐stained sections of formalin‐fixed skeletal muscle were characterized by the presence of numerous cytoplasmic vacuoles in myofibers that correlated with numerous, densely stained periodic acid–Schiff (PAS)‐positive cytoplasmic granules in frozen skeletal muscle. Additionally, there was mild‐to‐moderate myofiber degeneration and regeneration in H&E‐stained sections that were consistent with ongoing myofiber injury and repair. Relative to Pompe vehicle control mice, Pompe AT845‐treated mice (nine males and nine females per cohort) had lower heart weights at 3 × 10^14^ vg/kg and dose‐dependent reduction or absence of cardiac myofiber vacuolation at ≥ 3 × 10^13^ vg/kg. Additionally, AT845‐treated Pompe mice had dose‐dependent decreased incidence and severity or absence of myofiber vacuolation at ≥ 3 × 10^13^ vg/kg in skeletal muscle myofibers in H&E‐stained sections and in myofiber cytoplasmic glycogen accumulations in PAS‐stained sections (Fig 2). These microscopic observations were in agreement with the decrease in glycogen content measured by the biochemical assay (Fig 1D).

### Systemic delivery of AT845 leads to robust GAA synthesis and activity in skeletal and heart muscle of NHPs

To investigate the tolerability, potential toxicity, and safety pharmacology of AT845, we dosed juvenile cynomolgus NHPs at escalating doses via a single IV injection. A total of 22 NHPs with low anti‐AAV8‐neutralizing antibody titers (< 80 for controls, < 20 for AT845 dosed animals) were enrolled in the study. Six NHPs (three males and three females) per dose cohort were administered AT845 at doses of 6 × 10^13^ vg/kg (low dose), 2 × 10^14^ vg/kg (mid‐dose), or 5 × 10^14^ vg/kg (high dose). Given that wild‐type NHPs physiologically express GAA in all tissues, we used four control NHPs (two males and two females) injected with vehicle (Ringer's lactate solution with 0.01% poloxamer 188) as a reference for endogenous GAA activity. All NHPs were followed for 12 weeks.

**Figure 4:**
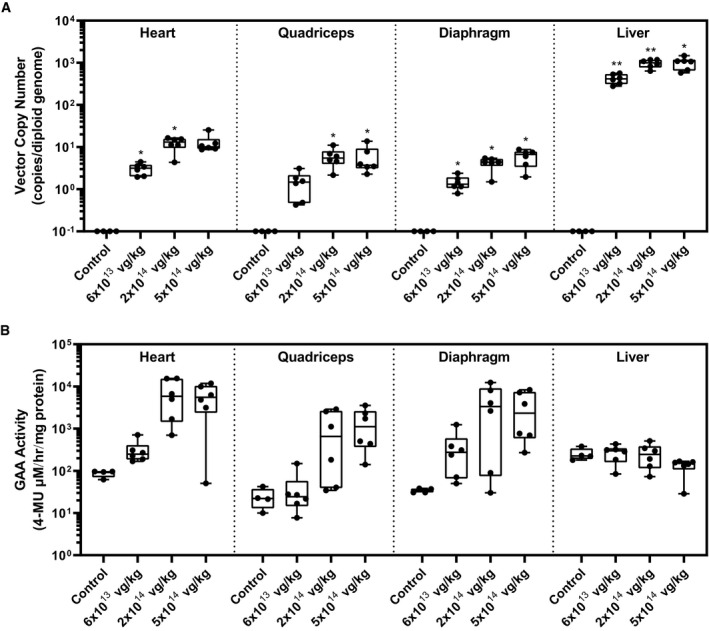
Dose‐dependent increase in transduction level and GAA activity in NHPs A, BEqual numbers of male and female cynomolgus monkeys were treated with vehicle control (*n* = 4) or escalating doses AT845 (*n* = 6) and followed for approximately 12 weeks. (A) Vector copy number and (B) GAA activity in heart, quadriceps, triceps, diaphragm, and liver. Statistical analysis: mixed‐effects analysis with Dunnett's test. Data information: Data are presented as box‐and‐whisker plots with Tukey whiskers that show minimum, median, and maximum values. Asterisks (*) indicate significant differences compared with vehicle control NHPs. *P < 0.05; and **P < 0.01.

**Figure EV2:**
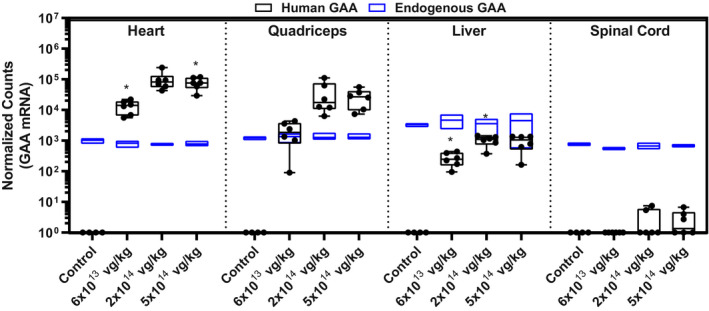
Dose‐dependent increases in vector‐derived human GAA mRNA in cardiac/skeletal muscles Equal numbers of male and female cynomolgus monkeys were treated with vehicle control (*N* = 4) or AT845 (*N* = 6) at the vector doses indicated and followed for approximately 12 weeks post‐injection. Endogenous GAA (blue) and human GAA (black) mRNA levels in heart, quadriceps, liver, and spinal cord. Statistical analysis: two‐way ANOVA, Dunnett's test. Data are presented as box‐and‐whisker plots with Tukey’s whiskers that show minimum, median, and maximum values. Asterisks (*) indicate significant differences compared with control (untreated NHPs). *P < 0.05.

Biodistribution of AT845 across treatment cohorts was assessed by measuring VCN in heart, quadriceps, diaphragm, liver, brain, spinal cord, sciatic nerve, kidney, lung, testes, and ovaries (Fig 4A and Appendix Fig S1). At the high dose of 5 × 10^14^ vg/kg, VCN was highest in the liver (998 ± 324 vg/dg) and lowest in the heart (12.6 ± 6.4 vg/dg), diaphragm (5.9 ± 2.6 vg/dg), and quadriceps (5.8 ± 4.3 vg/dg). VCN decreased in all tissues at decreasing doses, with heart, diaphragm, and quadriceps exhibiting 3.0 ± 1.0, 1.5 ± 0.6 and 1.5 ± 1.0 vg/dg at the lowest dose, respectively. Accordingly, GAA activity exhibited a dose‐dependent increase in the cardiac and skeletal muscle acquired from NHPs treated with AT845 compared with vehicle‐treated animals (Fig 4B and Appendix Fig S1). More specifically, GAA activity in NHPs treated at the highest dose was 60‐ to 100‐fold higher than values observed in vehicle‐treated animals in all tissues analyzed, which included the heart (*P* < 0.001), quadriceps (*P* < 0.001), and diaphragm (*P* < 0.01). GAA activity was detected at background endogenous levels in liver, central nervous system (CNS), and reproductive systems at all doses, confirming the tight restriction in gene expression provided by the MCK enhancer/promoter elements (Fig 4B and Appendix Fig S1). Furthermore, quantitative analysis of mRNA expression from the endogenous cynomolgus *GAA* gene and the AT845‐carried human *GAA* transgene by RNA sequencing showed a dose‐dependent increase in human *GAA* mRNA in heart and quadriceps, and levels below endogenous *GAA* mRNA in liver and spinal cord (Fig EV2). At the highest dose, AT845‐derived *GAA* mRNA accumulated at 93‐ and 16‐fold higher levels than endogenous *GAA* mRNA in the heart and quadriceps, respectively.

### Expression of human but not cynomolgus GAA elicits humoral immune response, inflammation, and elevation of cardiac biomarkers in NHPs

**Figure 5:**
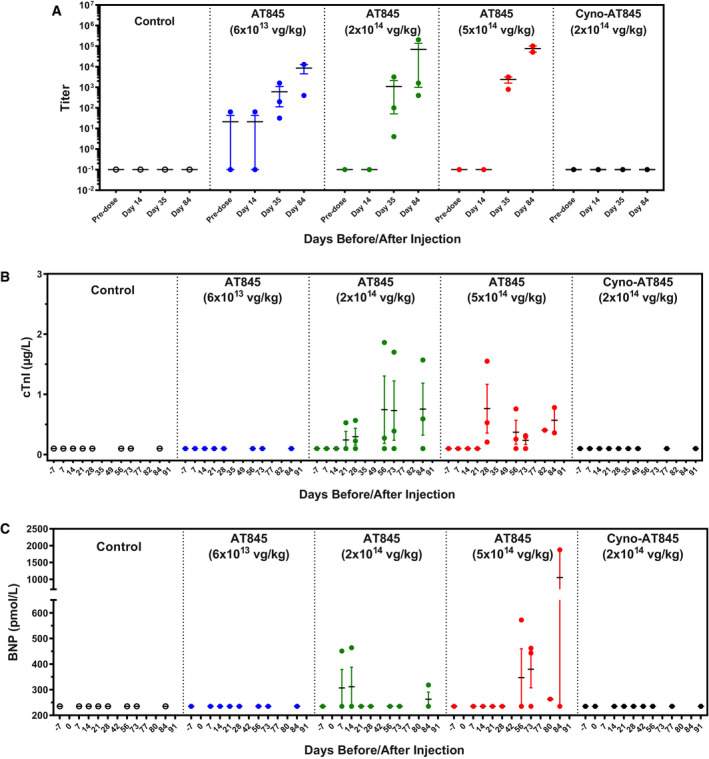
Anti‐GAA activity and cardiac biomarkers in male NHPs A–CMale cynomolgus monkeys were treated with vehicle control (*n* = 2), AT845 (*n* = 3) at 6 × 10^13^, 2 × 10^14^, or 5 × 10^14^ vg/kg, or cyno‐AT845 (*n* = 3) at 2 × 10^14^ vg/kg and followed for approximately 12 weeks. (A) Anti‐GAA antibody titer, (B) cardiac troponin I (cTnI), and (C) N‐terminal pro‐b‐type natriuretic peptide (BNP). Bars show mean and SEM.

Non‐human primates treated with AT845 at mid‐ and high doses showed multi‐organ toxicity that manifested as mononuclear cell infiltration and necrosis in heart, liver, skeletal muscle, dorsal root ganglia, and nerves by 12 weeks post‐dosing (histopathology results are described below). All NHPs treated at the high dose developed high levels (> 800) of anti‐GAA serum antibodies at 35 days post‐dosing, which increased at later time points (Fig 5A). Although our method cannot distinguish between anti‐human and anti‐cyno GAA antibodies, we assume that most or all of the humoral response was directed against the human enzyme. The highest dose was not tolerated and resulted in unscheduled deaths of one female on Day 79 due to declining clinical condition and one male on Day 82 due to echocardiographic findings in the absence of concerning clinical signs. Two animals in the low‐dose cohort showed low (< 80) anti‐GAA antibody titer before treatment, for unexplained reasons. When analyzed separately, female NHPs showed on average lower antibody titers than males at both Day 35 and Day 84, although the difference did not reach statistical significance. Biomarkers of cardiac abnormality correlated with the histopathological findings, and NHPs treated at the mid‐ and high dose showed a dose‐dependent elevation of cardiac troponin I (cTnI) and N‐terminal pro‐b‐type natriuretic peptide (NT‐proBNP; Fig 5B and C). Both cTnI and NT‐proBNP were undetectable in the serum of vehicle‐treated animals and in AT845‐treated animals before dosing, as well as post‐dosing in all animals treated at the lowest dose. Echocardiographic findings were generally noted in animals treated at the mid‐ and high doses, consisting of decreased left ventricular wall thickness, left ventricular dilation, valvular regurgitation, and arrhythmia (Appendix Table S2). Systolic dysfunction was observed in all three treatment groups (Appendix Table S2).

**Figure 6:**
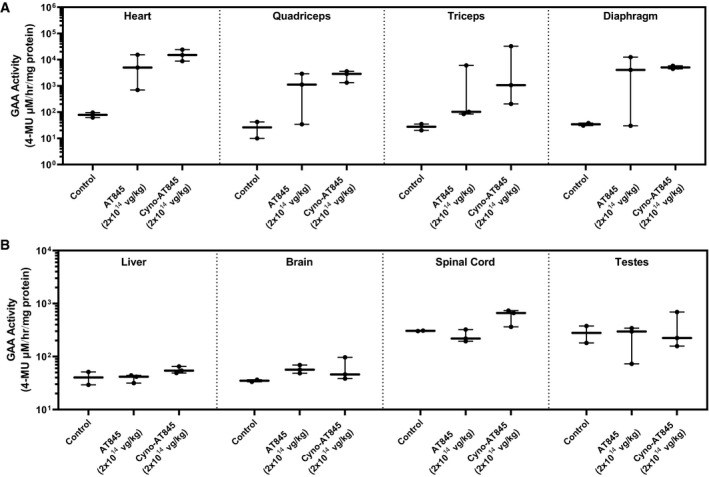
Human and cynomolgus GAA activity in NHPs A, BMale cynomolgus monkeys were treated with vehicle control (*n* = 2), AT845 (*n* = 3), or cyno‐AT845 (*n* = 3) at 2 × 10^14^ vg/kg and followed for approximately 12 weeks. GAA activity in (A) heart, quadriceps, triceps, and diaphragm, and (B) liver, brain, spinal cord, and testes. Data information: Data are presented as box‐and‐whisker plots with Tukey's whiskers that show minimum, median, and maximum values.

To determine whether the observed inflammatory and immune responses were caused by the xenogeneic human GAA protein, we dosed three additional male NHPs with a cynomolgus version of AT845 (cyno‐AT845) by a single IV infusion at a dose of 2 × 10^14^ vg/kg. The enzymatic activity of the transgenic GAA protein was similar or higher than that of the human version in animals treated at an equivalent dose of AT845, as measured in the heart, quadriceps, triceps, and diaphragm (Fig 6A). As expected, no significant elevation from baseline levels was observed in non‐muscle tissues (liver, brain, spinal cord, and testis; Fig 6B). Animals treated with cyno‐AT845 showed only minimal dorsal root ganglia and liver alterations (histopathology results are described below), and no elevation of cardiac biomarkers (Fig 5B and C). None of the animals raised detectable levels of anti‐GAA antibodies (Fig 5A), indicating that AAV‐driven overexpression of cynomolgus GAA does not break tolerance and does not raise a humoral immune response in immunocompetent NHPs during the 3‐month study duration.

### Processing of human GAA is incomplete in NHPs

**Figure 7:**
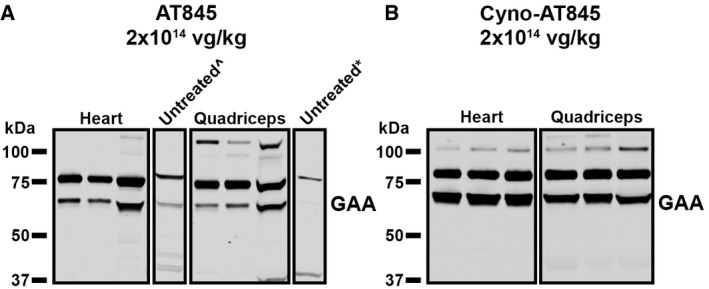
Species‐specific processing efficiency of GAA in cardiac/skeletal muscles A, BWestern blot analysis of heart and quadriceps lysates using anti‐GAA detects both the precursor GAA (110 kDa) and the mature lysosomal GAA (70–76 kDa) forms in muscle of NHPs treated with (A) AT845 (*n* = 3) or (B) cyno‐AT845 (*n* = 3) at 2 × 10^14^ vg/kg. Each tissue was analyzed in triplicate, and each respective symbol indicates use of a vehicle control heart tissue (^) or a vehicle control quadriceps tissue (*) (see also Appendix Table S3).

Typically, GAA is synthesized as an immature glycoprotein precursor (110 kDa) in the endoplasmic reticulum and undergoes a series of proteolytic and *N*‐glycan processing events to yield an intermediate (95 kDa) and two lysosomal (76 and 70 kDa) isoforms, the latter represent the mature species of the enzyme and are considered to be essential for GAA activation (Wisselaar *et al*, 1993; Moreland *et al*, 2005). To characterize processing of human and cynomolgus GAA proteins, we carried out a GAA‐specific (anti‐human or anti‐cynomolgus) immunoblot analysis on the cardiac and skeletal muscle samples collected 12 weeks after treatment. In AT845‐treated NHPs, the human GAA transgene was processed in both heart and quadriceps primarily to the 76 kDa isoform, with lower levels of the 70 kDa isoform (Fig 7A). As a result, the ratio between the 76 and 70 kDa isoforms was ~10:1 (Appendix Table S3). We also detected a residual 110 kDa isoform in the quadriceps, suggesting some incomplete processing of the exogenous GAA. Conversely, in the heart and quadriceps of NHPs treated with cyno‐AT845, we detected little or no 110 kDa precursor or 95 kDa intermediate that was predominantly converted into an almost equal ratio (~1:1) of the 76/70 kDa mature isoforms, indicating that enzyme processing was not saturated by GAA overexpression (Fig 7B and Appendix Table S3). Taken together with the GAA activity data, these results suggest that the enzymatic activity of GAA derived from AT845 and cyno‐AT845 administration correlates with the increased levels of the mature, lysosomal isoforms of GAA and that processing of human GAA may be incomplete in NHPs.

### NHP histopathology

**Figure EV3:**
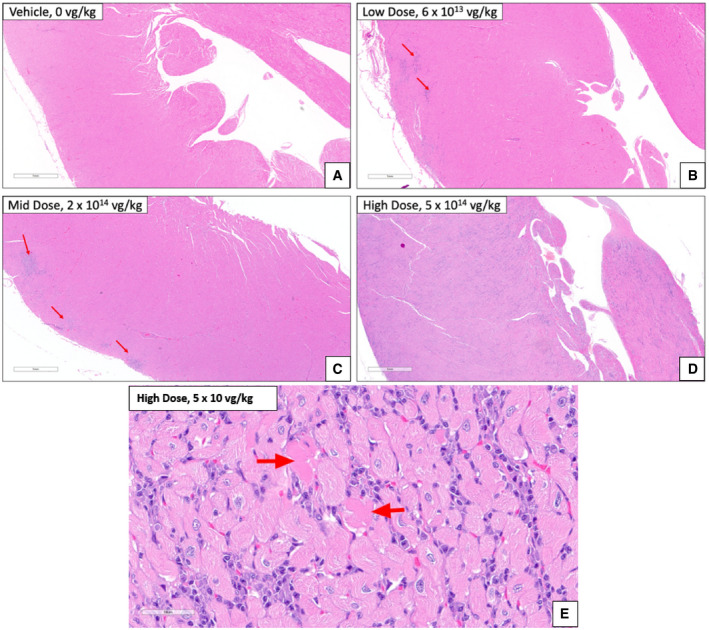
Cardiac histopathological findings in NHPs treated with AT845 (H&E staining) A–DArrows indicate tissue basophilia (blue staining areas) at the low and mid‐dose. The inflammation associated with the low and mid‐dose was multifocal and ranges from minimal to mild. At high dose, the inflammation is diffuse and moderate. Scale bar = 1 mm.EDegeneration of cardiac myofibers in cynomolgus macaque treated with AT845 at 5 × 10^14^ vg/kg, designated by arrows. Inflammation and cardiac myofiber degeneration were not present in AT845‐cyno‐dosed macaques. Scale bar = 50 µm.

**Figure EV4:**
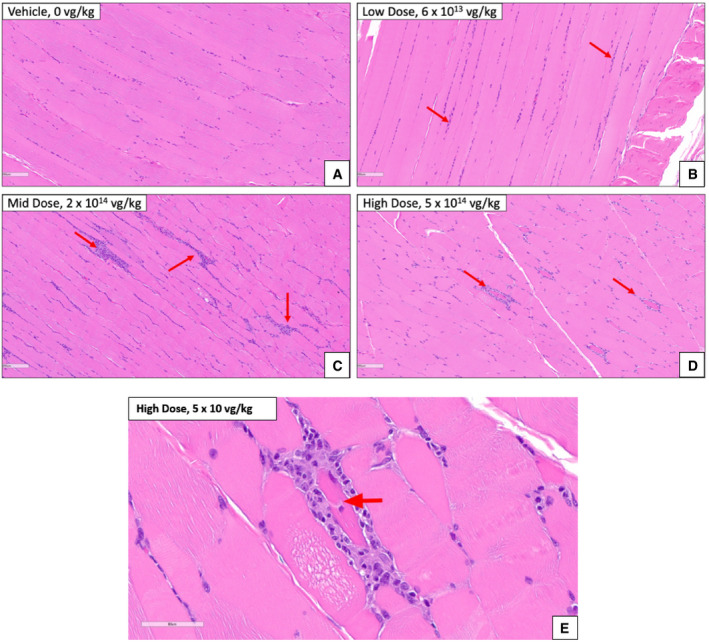
Skeletal muscle (quadriceps) histopathological findings in NHPs treated with AT845 (H&E staining) A–DArrows indicate multiple foci of basophilia (mixed cell inflammation) between myofibers (interstitial). The dark pink‐stained cells at the center of the inflammation in the high‐dose male are degenerated myofibers. Scale bar = 200 µm.EDegeneration of skeletal myofiber in cynomolgus macaque treated with AT845 at 5 × 10^14^ vg/kg, designated by an arrow. This was not present in AT845‐cyno‐dosed macaques. Scale bar = 60 µm.

**Figure EV5:**
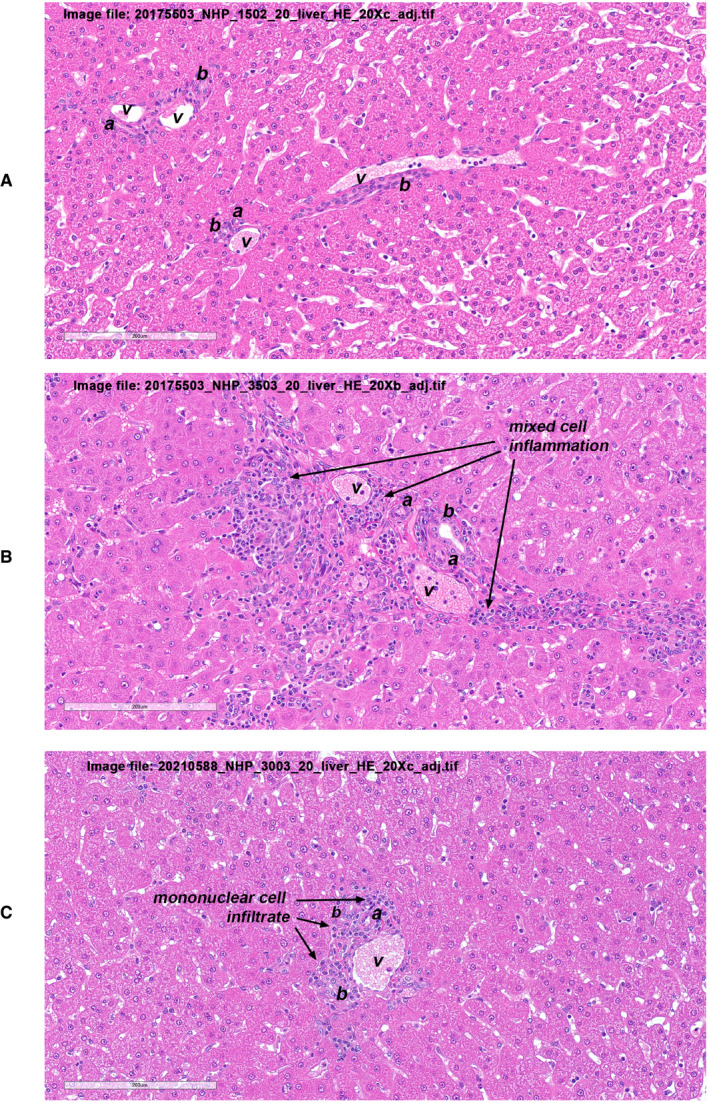
Liver histopathological findings in NHPs treated with AT845 (H&E staining) A–CMicroscopic observations in liver sections from a control cynomolgus macaque (A), a cynomolgus macaque treated with AT845 at 2 × 10^14^ vg/kg (B), and a cynomolgus macaque treated with cyno‐AT845 at 2 × 10^14^ vg/kg (C). Hepatic portal triads are designated by portal veins (v); portal arteries (a); and bile ducts (b). Panel A represents a normal hepatic portal triad. In Panel B, portal triad structures are surrounded by a moderate mixed cell inflammatory infiltrate consisting of lymphocytes, plasma cells, and histiocytes. In Panel (C), portal triad structures are surrounded by a minimal mononuclear cell infiltrate consisting primarily of histiocytes. Scale bar = 200 µm.

**Figure EV6:**
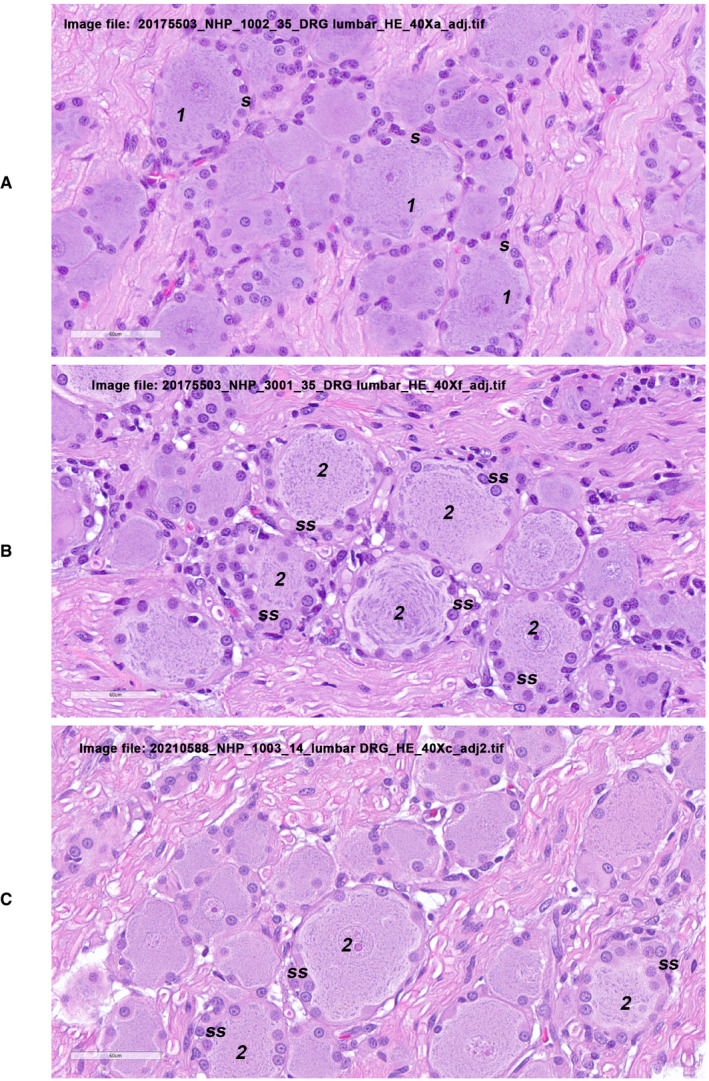
Dorsal root ganglia histopathological findings in NHPs treated with AT845 (H&E staining) A–CMicroscopic observations in H&E‐stained dorsal root ganglion sections from a control cynomolgus macaque (A), a cynomolgus macaque treated with AT845 at 2 × 10^14^ vg/kg (B), and a cynomolgus macaque treated with cyno‐AT845 at 2 × 10^14^ vg/kg (C). Neurons labeled “1” in Panel A are surrounded by a single layer of normal satellite cells (s), each characterized by scant acidophilic‐to‐amphophilic cytoplasm and a round‐to‐oval basophilic nucleus with finely dispersed chromatin and single small nucleolus. Neurons labeled “2” in Panels (B) and (C) are surrounded by satellite cells that are increased in number and size (ss), arranged in one to two layers, and characterized by moderate amounts of cytoplasm. Scale bar = 60 µm.

Juvenile cynomolgus macaques were administered AT845 via a single intravenous dose at 6 × 10^13^, 2 × 10^14^, or 5 × 10^14^ vg/kg. The highest dose was not tolerated and resulted in unscheduled deaths of one female on Day 79 and one male on Day 82. There were AT845‐related microscopic changes consisting of mixed inflammation in multiple tissues, including heart, skeletal muscles, smooth muscles, and liver at doses ≥ 2 × 10^14^ vg/kg (Figs EV3, EV4, EV5). Additionally, there were glial cell hypertrophy and hyperplasia and individual neuronal degeneration of dorsal root ganglia (Fig EV6). The test article‐related microscopic findings associated with cyno‐AT845 consisted of minimal‐to‐mild glial cell hypertrophy/hyperplasia of the dorsal root ganglia accompanied by mononuclear cell infiltrates, and individual neuronal degeneration. In the liver, there were minimal mononuclear‐to‐mixed cell infiltrates. Of uncertain association with cyno‐AT845 were mononuclear cell infiltrates in the prostate gland, heart, and kidneys.

## Discussion

Enzyme replacement therapy, based on systemic administration of recombinant human GAA, is currently the only available treatment for PD (Kishnani & Beckemeyer, 2014). Although beneficial for most patients, the efficacy of ERT is limited by the pharmacokinetics of the recombinant enzyme, which requires frequent administrations, and the formation of antibody responses that can interfere with the uptake of GAA in the cardiac and skeletal muscle tissues. In addition, immunogenicity of the recombinant enzyme represents a formidable challenge in CRIM‐negative patients, for whom therapeutic efficacy is limited even at higher doses and with more intense administration schedules (Kishnani *et al*, 2007; van der Ploeg *et al*, 2010; Kishnani & Beckemeyer, 2014; Schoser *et al*, 2017). Gene therapy protocols based on delivery to the liver of AAVs encoding a wild‐type or an N‐terminal modified, secretable GAA have been proposed as a potential alternative to ERT (Han *et al*, 2017; Puzzo *et al*, 2017). The approach represents an internal organ‐driven ERT, with the advantage of better pharmacokinetics and increased bioavailability of an endogenously processed and potentially less immunogenic enzyme. Interestingly, expression of GAA in the liver was shown to induce a tolerogenic response that reduces or eradicates anti‐GAA antibody responses in *Gaa* ^−/−^ mice (Dobrzynski *et al*, 2004; Sun *et al*, 2007; Zhang *et al*, 2012; Doerfler *et al*, 2016; Han *et al*, 2017). It remains unclear, however, whether liver‐induced tolerance would occur in species other than rodents and, most importantly, in CRIM‐negative patients (Colella & Mingozzi, 2019).

Uptake of exogenous GAA by the muscle tissue remains a limit of both ERT and systemic enzyme delivery by transgenic hepatocytes. We therefore developed AT845, an AAV8 vector designed to express GAA in skeletal muscles and heart, and evaluated its performance in a mouse model of PD and in a toxicology study in NHPs. The GAA expression cassette of AT845, driven by an MCK promoter/enhancer combination, was designed to allow robust, tissue‐restricted expression of wild‐type enzyme within muscle fibers and cardiomyocytes, thus relying on their physiological machinery for enzyme processing and lysosomal targeting. Systemic administration of AT845 in *Gaa* ^−/−^ mice led to a dose‐dependent increase in GAA expression and activity, glycogen clearance in skeletal and cardiac muscles, and functional improvement. Reduction in glycogen storage was obtained with doses of AT845 as low as 3 × 10^13^ vg/kg, while doses in the order of 10^14^ vg/kg, which are known to provide efficient and durable AAV delivery to the majority of muscle fibers in humans, led to almost complete normalization of glycogen levels in the skeletal muscle and heart as early as 3 months after administration. Quantitation of GAA transcript, protein level, and enzyme activity showed that AT845 allows accumulation of active enzyme in the muscle tissue at supraphysiological levels that are required for rapid clearance of glycogen storages in the mouse model of PD, as also shown in studies describing a liver‐directed approach (Han *et al*, 2017; Puzzo *et al*, 2017). Importantly, supraphysiological GAA levels did not result in glycogen depletion below physiological levels, even at the highest dose of AT845. Glycogen levels, which are inherently higher in diaphragm compared with heart or skeletal muscle in this mouse model (Keeler *et al*, 2019; Han *et al*, 2020), can fluctuate up to 100% daily (Gomes *et al*, 2009). High glycogen levels in some mice may reflect time of euthanasia.

When tested in NHPs, AT845 caused a dose‐dependent increase in GAA activity in skeletal muscle and heart, which was < fivefold at the lowest dose and plateaued at 10‐ to 50‐fold higher than the physiological levels at doses ≥ 2 × 10^14^ vg/kg. Such an increase in GAA activity is significantly more robust than that obtained in NHPs treated with a liver‐directed AAV vector, albeit at a lower and potentially less toxic dose (Puzzo *et al*, 2017). The GAA levels obtained in NHPs with AT845 predict a wide therapeutic window in a dose range that, based on existing clinical evidence, is likely to cause minimal toxicity in humans. Restriction of gene expression was also confirmed in NHPs, where GAA activity remained at physiological levels in the liver at all doses despite a VCN in the order of 200–1,400 vg/dg.

The highest doses of AT845 (≥ 2 × 10^14^ vg/kg) caused anti‐GAA humoral immune response and inflammation in various tissues, elevation of cardiac biomarkers, and echocardiogram abnormalities in NHPs. On the contrary, a vector expressing the cynomolgus macaque GAA, and otherwise identical to AT845, did not cause multi‐organ inflammation nor immune response at a dose of 2 × 10^14^ vg/kg. Of note, cyno‐AT845 induced GAA activity at levels comparable or higher than those achieved with the human version of AT845 at the same dose. These results suggest that the inflammatory reactions observed in NHPs treated with AT845 were due to an anti‐GAA xenogeneic immune response rather than GAA overexpression. Interestingly, degeneration of dorsal root ganglia neurons was still observed in animals treated with cyno‐AT845, suggesting that this effect could be the result of transduction and protein expression, as proposed by recently published studies (Hordeaux *et al*, 2020a, 2020b). However, in our studies the vector copy number in nervous tissues was very low and GAA activity levels in these tissues were not above endogenous. We hypothesize that the dorsal root ganglia changes in the neurons and satellite cells may be related to the presence of vector capsids.

The dramatic xenogeneic reaction observed in NHPs was somewhat surprising, since human proteins are regularly expressed in toxicology studies of AAV‐based gene therapies with little immune consequences. In particular, toxicity was apparently not observed in NHPs treated with an AAV8‐GAA vector designed for liver‐directed gene therapy for Pompe disease (Puzzo *et al*, 2017). This may be due to the relatively low AAV vector dose used in the study (2 × 10^12^ vg/kg) or to a tolerogenic response induced by liver expression. The human (https://www.uniprot.org/uniprot/P10253) and cynomolgus macaque (https://www.uniprot.org/uniprot/A0A2K5VE94) GAA proteins share a 95% sequence identity, differing in only 45 of 952 amino acids, 17 of which are concentrated in the 205 amino acids of the N‐terminal catalytic domain. This relatively small number of differences further emphasizes the remarkable immunogenicity of GAA and induces a note of caution in the prospective treatment of CRIM‐negative patients by gene therapy, which will likely require some form of immune suppression or tolerization regimen. Nonetheless, the NHP studies may predict the safety of expressing even high levels of the enzyme in the muscle and heart of CRIM‐positive patients, who are tolerant to human GAA.

An interesting corollary of our studies is the demonstration that processing of GAA from the 110 kDa precursor to the mature, active 76/70 kDa isoforms is incomplete in NHP skeletal and cardiac muscle. Inefficient processing, and subsequent reduced accumulation of the active enzyme isoforms in the lysosome, may have contributed to the anti‐human GAA immune response in NHPs. Should human GAA processing be defective also in rodents, it may have reduced the activity of AAV‐directed GAA in our GAA‐deficient mouse study. Overall, our results point to limitations in predicting efficacious doses of gene therapy products in murine models of lysosomal storage disorders and raise the issue of xenogeneic responses as confounding factors in toxicology studies utilizing immunocompetent non‐human species.

This study shows that AT845 leads to a robust, dose‐dependent increase in GAA activity at therapeutic, supraphysiological levels in both mice and NHPs, resulting in glycogen clearance and functional improvement in GAA‐deficient mice. Lack of immune responses and toxicity in NHPs tolerant to the native enzyme supports the progression of AT845 to clinical testing. A Phase I/II, international, open‐label, ascending dose clinical trial aimed at assessing safety and efficacy of AT845 in CRIM‐positive LOPD patients (Clinical trial identifier: NCT04174105) is activated in the United States and is currently recruiting patients.

## Materials and Methods

### Vector structure and production

The rAAV8‐eMCK‐hGAA vector (AT845) is a single‐stranded recombinant AAV containing a human *GAA* cDNA, codon‐optimized to reduce its CpG content, under the control of the murine MCK promoter preceded by its short enhancer (Johnson *et al*, 1989; Wang *et al*, 2008). The vector contains the small intron and late polyadenylation signal sequence of the simian virus 40 (SV40, Fig EV1). The vector was produced by cotransfection of the vector plasmid and a single helper construct expressing AAV Rep and serotype‐8 Cap and adenovirus helper functions in HEK293 cells in suspension culture in bioreactors, following an Audentes proprietary manufacturing process. Vector batches were titrated by digital droplet PCR, and titer was expressed as infectious genomes per volume unit (ig/ml). A cynomolgus version of AT845 (cyno‐AT845) was built using a cynomolgus *GAA* cDNA instead of human *GAA* cDNA in an otherwise identical vector and produced using the same process.

### Animals

All protocols involving the use of animals were approved by the Institutional Animal Care and Use Committee (IACUC) of Charles River Laboratories at Mattawan, MI (mice), or at Reno, NV (NHPs). All experiments were performed in accordance with relevant guidelines and regulations. Mice used in this study were B6;129‐Gaa^tm1Rabn^/J (*Gaa* ^−/−^) from the Jackson Laboratory (Bar Harbor, ME, USA), a *Gaa* KO strain carrying a targeted deletion of exon 6, and wild‐type littermates of the KO mouse model (B6;129). The NHPs were juvenile Chinese cynomolgus macaques aged 28–38 months sourced from different vendors. The NHPs were screened for the absence of anti‐AAV8‐neutralizing antibodies with a cutoff titer of < 20.

### Mouse minimum efficacious dose study design

Eighteen wild‐type (WT) and 72 *Gaa* ^−/−^ mice were used in this study, which consisted of five groups: vehicle‐treated WT; vehicle‐treated *Gaa* ^−/−^, and AT845‐treated *Gaa* ^−/−^. Vehicle‐treated animals were injected with Ringer's lactate containing 0.01% Pluronic. All animals were treated on Study Day 1 with two bolus injections in the tail vein 30–60 min apart at a total dose of 3 × 10^13^, 1 × 10^14^, or 3 × 10^14^ vg/kg, and analyzed 3 months post‐injection. Cohort 1 consisted of five males and five females for each group and was intended for clinical pathology and histopathology evaluations. Cohort 2 consisted of four males and four females for each group and was designated for bioanalytical and biodistribution evaluations.

### NHP good laboratory practice toxicology study design

For the human AT845 toxicology study, 22 NHPs were divided into four groups: control group (two males, two females) was injected with Ringer's lactate containing 0.01% Pluronic, while the other three groups (three males, three females each) received AT845 at a dose of 6 × 10^13^, 2 × 10^14^, or 5 × 10^14^ vg/kg. Animals were dosed via a single IV infusion in a suitable peripheral vein on Study Day 1 using a calibrated infusion pump (target infusion time: 2 h) and analyzed 3 months after dosing. For the cynomolgus‐specific AT845 toxicology study, three males received cyno‐AT845 at a dose of 2 × 10^14^ vg/kg with the same modalities and were analyzed 3 months after dosing.

### Vector copy number

Vector copy number was quantified using a qualified real‐time qPCR assay with primers and probes designed on the human or cynomolgus codon‐optimized cDNA sequence, using a linearized AT845 vector plasmid as reference standard. Each 20 μl qPCR contained 100 ng sample gDNA. A positive signal was defined as detection of ≥ 50 vector copies of hGAAco1 per qPCR. Samples with < 50 vector copies per reaction were reported as below the limit of quantification. A standard curve was prepared with AT845 vector plasmid in a background matrix of 200 ng/μl mouse or cynomolgus gDNA isolated from liver. Each DNA sample was run in triplicate, including one replicate spiked with 100 copies of AT845 vector‐positive control to assess PCR inhibition.

### GAA ELISA

An ELISA method for the quantitative measurement of the human or cynomolgus GAA proteins in mouse or NHP tissue extracts was developed and qualified at Audentes Therapeutics. Briefly, 96‐well ELISA plates were coated overnight with proprietary anti‐human or anti‐cynomolgus GAA antibodies at 2 µg/ml. The coated plates were blocked, protein lysates and control standards were immobilized to the plates by a 2‐h incubation under gentle agitation, and plates were washed to remove unbound material. Biotinylated goat polyclonal anti‐GAA detection antibody (Poly0619) solution at 0.5 µg/ml was added to the wells and incubated for 1 h. After washing, 1 µg/ml streptavidin poly‐HRP detection solution was added to the wells and incubated for 20 min under gentle shaking, followed by washing, the addition of the TMB substrate, and incubation for 15 min. The reaction was stopped by the addition of stop solution, and the intensity of the blue color was measured at 450 nm with a background correction at 570 nm. A weighted 4‐parameter logistic model [1/Sqrt(Conc)] was applied to fit the standard curve and used to back‐calculate the GAA concentrations (pg/ml) in the samples and controls based on the measured absorbance values. The concentration of GAA for each sample was multiplied by the sample dilution factor to determine the adjusted sample GAA concentration, expressed as ng/ml. Total protein in tissue lysate was quantitated with the BCA assay using BSA standards. The adjusted GAA concentrations for each sample were divided by the corresponding adjusted protein concentration (in mg) to obtain ng of GAA per milligram of tissue.

### GAA enzyme activity

To measure GAA enzyme activity, sera or supernatants from tissue homogenates were incubated with 4‐methylumbelliferyl α‐d‐glucopyranoside (4‐MUG), a fluorogenic substrate that is hydrolyzed to 4‐methylumbelliferone (4‐MU) by GAA. The reaction was stopped with an alkaline buffer, in which 4‐MU fluoresces at a different wavelength from the unhydrolyzed 4‐MUG. 4‐MU (λex = 360 nm and λem = 460 nm) was detected with a fluorescence plate reader. A 4‐parameter logistic fit was applied to the standard curve and used to calculate 4‐MU concentrations (in μM) for samples and controls based on the measured relative fluorescence unit values. The 4‐MU concentrations (in μM) were multiplied by the sample dilution factor to determine the adjusted 4‐MU concentrations (in μM). Protein was quantitated with the BCA assay using albumin standards. The 4‐MU concentrations (in μM) were divided by the protein concentration (mg/ml), and the result was divided by the length of the assay (1 h) to express GAA activity as μmol 4‐MU/hour/mg protein.

### Glycogen levels

To measure glycogen levels, 250–300 µl of supernatant from tissue homogenates was transferred to a 1.5‐ml tube, placed in boiling water for 10 min, and then centrifuged. The supernatant was collected and transferred to prelabeled cryovials for glycogen analysis. In the glycogen assay (Abcam, ab65620), glucoamylase, an alpha‐glucosidase digestive enzyme, hydrolyzes the glycogen to glucose, which is then specifically oxidized to produce a product that reacts with OxiRed probe to generate fluorescence, which is detected in a plate reader (λex = 535 nm and λem = 587 nm).

### Microscopic assessment of glass slides and creation of image files for publication

Tissues were collected and preserved in 10% neutral buffered formalin, embedded in paraffin, sectioned, mounted on glass slides, and stained with H&E. Tissue slides were evaluated microscopically by a board‐certified veterinary pathologist. A peer review of the slides was conducted by a second board‐certified veterinary pathologist.

Whole glass slide imaging was performed with a brightfield slide scanner (Aperio AT2 with ScanScope Console version 102.0.75 and eSlide Manager version 12.4.3.7006; Leica Biosystems). The resolution of each whole slide image (eSlide) was 0.253 microns per pixel, and the apparent magnification was 40×.

eSlides were reviewed, and images of specific regions were captured in TIF format utilizing digital image viewing software (Aperio ImageScope version 12.4.3.5008; Leica Biosystems).

### Histochemistry and GAA immunohistochemistry

Muscle cryosections were cut (4–5 μm), placed onto slides, and air‐dried for 20 min. H&E and PAS stains were performed according to established protocols. For GAA immunohistochemistry, samples were fixed in 100% ice‐cold methanol for 15 min, washed in TBST (TBS containing 0.1% Tween 20) for 5 min, blocked in 2.5% goat serum at room temperature for 30 min, and incubated overnight at 4°C with an anti‐GAA rabbit antibody (Abcam, ab137068, 0.621 mg/ml) diluted 1:50 in TBST. Slides were washed three times for 10 min in TBST and incubated for 1 h at room temperature with a 1:400 dilution of Alexa Fluor 488‐conjugated goat anti‐rabbit IgG (Invitrogen, A11008) in TBST as a secondary antibody mix. Slides were then washed three times for 10 min in TBST, rinsed once in deionized H_2_O, and mounted in PermaFluor.

### Western blot analysis

Protein samples were resuspended in 20 μl of sodium dodecyl sulfate protein sample buffer followed by resolving of the protein and Western transfer using standard protocols (Invitrogen). GAA protein in the sample was detected using specific antibodies against GAA at a final concentration of 1 µg/ml followed by incubation with appropriate infrared dye‐tagged (IR680 and IR800) secondary antibody (1:5000 dilution) and scanning with an Odyssey infrared scanner (LI‐COR Biosciences, Lincoln, NE). Approximately, 20 μg tissue homogenate and 5 ng purified recombinant hGAA were used for analysis. Human GAA proteins were detected using rabbit polyclonal anti‐GAA antibody (BosterBio, A01548‐1) and rabbit monoclonal anti‐GAA antibody (Abcam, ab137068).

### Anti‐GAA antibody analysis

Briefly, ELISA plates were coated with rhGAA enzyme overnight and then blocked with StabilBlock solution for 1 h at room temperature. Samples and controls were diluted 1:20 and incubated for 1 h at room temperature. After a washing step, Protein G (HRP) was added, and the plates were incubated at room temperature for 30 min. 3,3′,5,5′‐Tetramethylbenzidine substrate (TMB) was then added for color development, followed by addition of a stop reagent for the TMB substrate. Plates were read at 450 nm with background correction at 650 nm.

### Troponin I assay

IMMULITE 2000 Troponin I is a solid‐phase, enzyme‐labeled chemiluminescent immunometric assay. The solid phase (bead) was coated with monoclonal murine anti‐troponin I antibody. The liquid phase consisted of alkaline phosphatase (bovine calf intestine) conjugated to polyclonal goat anti‐troponin I antibody. The sample and the reagent were incubated together with the coated beads for 30 min. During this time, troponin I in the sample formed the antibody sandwich complex with monoclonal murine anti‐troponin I antibody on the bead and the enzyme‐conjugated polyclonal goat anti‐troponin I antibody in the reagent. Unbound sample and enzyme conjugate were then removed by centrifugal washes. Finally, chemiluminescent substrate was added to the reaction tube containing the bead and the signal was generated in proportion to the bound enzyme.

### NT‐proBNP assay

The NT‐proBNP assay is based on the competitive binding of the unlabeled peptide present in samples or standards with the horseradish peroxidase‐labeled peptide (tracer) for the NT‐proBNP (8–29)‐specific antibody in multi‐well plates. The concentration of the tracer and capture antibody was kept constant in all wells, while that of test samples or standard was progressively increased. After removal of unbound tracer by washing, TMB was added to the wells. The amount of HRP‐labeled tracer bound to the microplate was quantitated on a standard ELISA reader. The intensity of developed color was inversely proportional to the amount of NT‐proBNP immunoreactivity present in the tested samples. A standard curve was plotted from the values measured and the concentrations of NT‐proBNP in the samples calculated from this curve. The ElA assay plate was read using the Molecular Devices VersaMax Plate Reader and SoftMax® Pro Software GxP v6.4. The LLOQ for NT‐proBNP was 235 pmol/l.

### Grip response

Grip response was measured in all mice at predose (Day ‐4 or ‐5) and at Weeks 6 and 12 using an inverted screen, as previously described (Kim *et al*, 2010). The total time that the animal remained hanging on the screen was recorded, with a maximum testing duration of 60 s. If the animal climbed to the top of the screen, the trial was terminated, and a score of 60 s was assigned.

### Echocardiography

Transthoracic echocardiography was performed on sedated animals in a darkened and quiet room with the NHP positioned in right and then left lateral recumbency on a purpose‐designed echocardiographic positioning table, which allowed placement of the ultrasound transducer on the dependent side of the thorax. The animal was shaved (approximately 2″ × 2″ on both the left and right axillary regions) to facilitate imaging when needed. Ultrasound gel was used to provide contact with the surface of the animal. A concurrent electrocardiogram was obtained at the time of the echocardiogram for timing of cardiac events. All examinations were performed by a board‐certified veterinary cardiologist, and offline analysis was performed on a dedicated computer using EchoPac software (V202). The echocardiograms were obtained with a GE Vivid iq Premium echocardiographic recorder (SW version/raw data acquisition device version: 1.2.4.5373, station ID: Viq‐6017687WX0). Ultrasound model configurations are as follows: GE Vivid iq R2 Premium system console with Auto 2D EF, 2D strain imaging, EchoPac Workstation, EchoPac software (V202). 12S‐RS phased array sector probe was ranged from 4.5‐ to 12.0‐MHZ scanner frequency. Two‐dimensional, M‐mode, spectral Doppler, color flow Doppler, tissue Doppler imaging, tissue velocity imaging, and speckle tracking echocardiographic measurements were performed offline after image acquisition. Offline measurements were performed using EchoPac software (V202) by the same board‐certified veterinary cardiologist.

### Statistics

Sample sizes were based on the study design guidelines specific for preclinical studies for gene replacement therapies. The total number of animals used, as well as the group size and the number of groups, was considered to be the minimum required to properly characterize the effects of AT845 as required to support regulatory submissions and was designed such that it does not require an unnecessary number of animals to accomplish its objectives.

For mouse studies, animals were randomized into treatment groups using a standard, by weight, measured value randomization procedure. Sample collection from mice was done in an alternating fashion (one animal per sex from each dose group, then repeating) to reduce handling and time biases. Blinding was performed when practical and allowed by experimental design. For the mouse PAS tissue analysis, the pathologist conducting the microscopic evaluation was blinded to the identity and treatment of the animal represented on the slide. For NHP and mouse H&E staining analyses, no steps were taken.

All data are expressed as box‐and‐whisker plots with Tukey's whiskers that show minimum, median, and maximum values, unless stated otherwise. Statistical analyses were conducted using GraphPad Prism version 8.4.3 (GraphPad Software, La Jolla, CA) or SAS/STAT version 14.1 (SAS Institute, Cary, NC). Differences among multiple populations were statistically significant at *P ≤ 0.05, **P ≤ 0.01, ***P ≤ 0.001, or ****P ≤ 0.0001, and calculated using a two‐way ANOVA, Dunnett's test.

## Author contributions

ME, CHV, and FM designed the study, analyzed the data, and wrote the manuscript. JC designed the primate study, analyzed the data, and reviewed the manuscript. JH, PP, and CS performed and analyzed experiments and reviewed the manuscript. JB, CF, MWL, HM, and MJP analyzed the data and reviewed the manuscript.

## Supporting information



Appendix

Source Data for Figure 1

Source Data for Figure 3

## Data Availability

This study includes no data deposited in external repositories.
