# Supplementary material for: Muscle‐directed gene therapy corrects Pompe disease and uncovers species‐specific GAA immunogenicity
Source: EMBO Mol Med. 2021 Dec 1;14(1):e13968. doi: 10.15252/emmm.202113968 (PMC8749482; doi:10.15252/emmm.202113968)
Supplement: Supplementary file 1 — Appendix [file EMMM-14-e13968-s004.pdf]

Appendix

Table of Contents

Appendix Figure S1. Dose-dependent increases in transduction efficiency and GAA activity in nonhuman primates.

Appendix Figure S2. Cardiac biomarkers in female NHPs.

Appendix Table S1. Glycogen levels in the muscle of wild-type and Gaa-/- mice.

Appendix Table S2. Echocardiographic findings in NHPs.

Appendix Table S3. Relative quantification of the percentage of each GAA Western blot band based on their signal intensity.

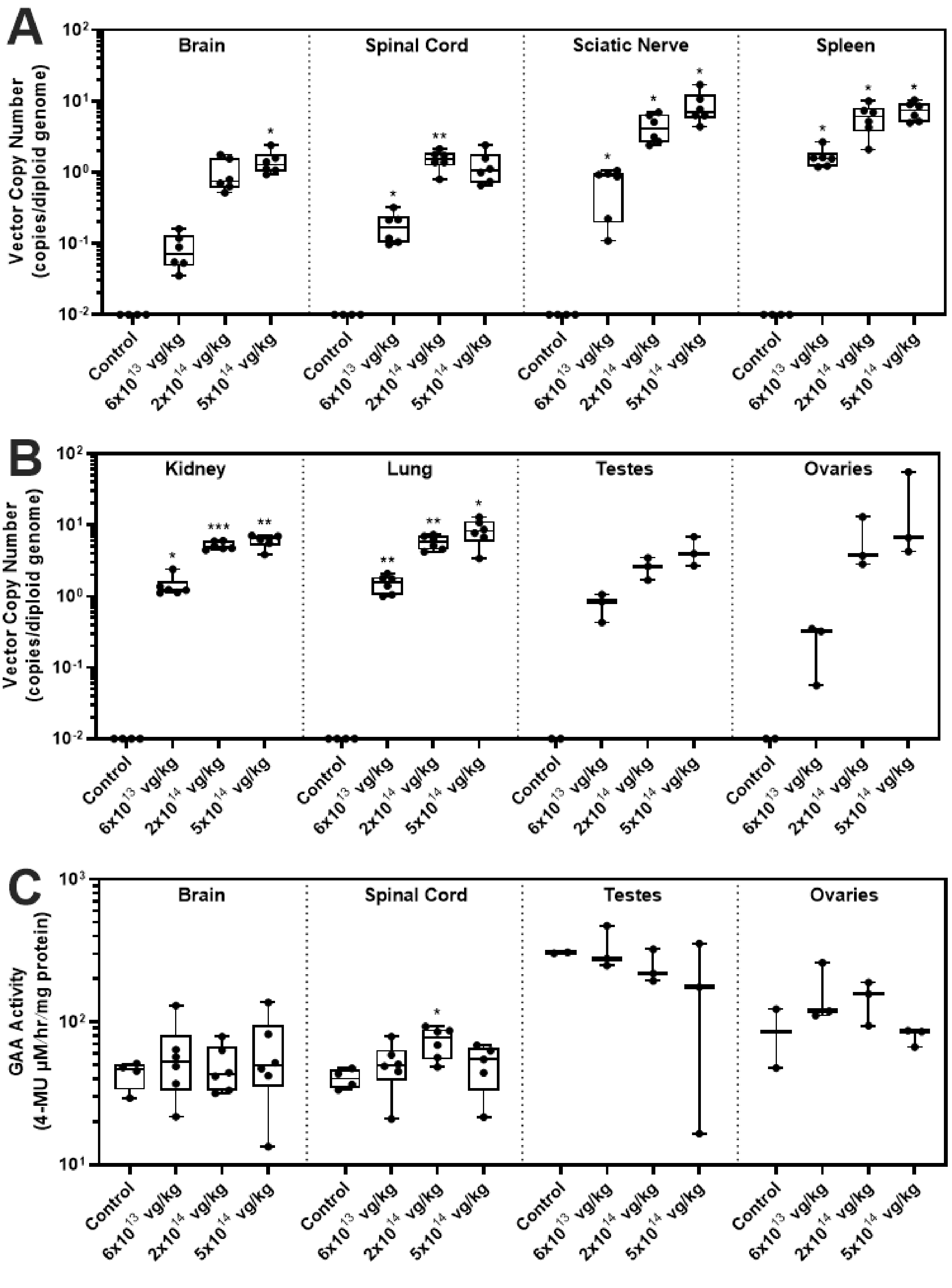
A
B
C
Brain
Spinal Cord
Sciatic Nerve
Spleen
Kidney
Lung
Testes
Ovaries
Vector Copy Number
(copies/diploid genome)
GAA Activity
(4-MU µM/hr/mg protein)
Control
6x10^13 vg/kg
2x10^14 vg/kg
5x10^14 vg/kg

Appendix Figure S1. Dose-dependent increases in transduction efficiency and GAA activity in nonhuman primates. Equal numbers of male and female cynomolgus monkeys were treated with vehicle control (N=4) or AT845 (N=6) at the vector doses indicated and followed for approximately 12 weeks. (A, B) Vector copy number in brain, spinal cord, sciatic nerve, spleen, kidney, lung, testes, and ovaries. (C) GAA activity in brain, spinal cord, testes, and ovaries. Statistical analysis: two-way ANOVA, Dunnett's test. Data are presented as box-and-whisker plots with Tukey whiskers that show minimum, median, and maximum. Asterisks (*) indicate significant differences compared with control (untreated NHPs). *, $P<0.05$; **, $P<0.01$; ***, $P<0.001$.

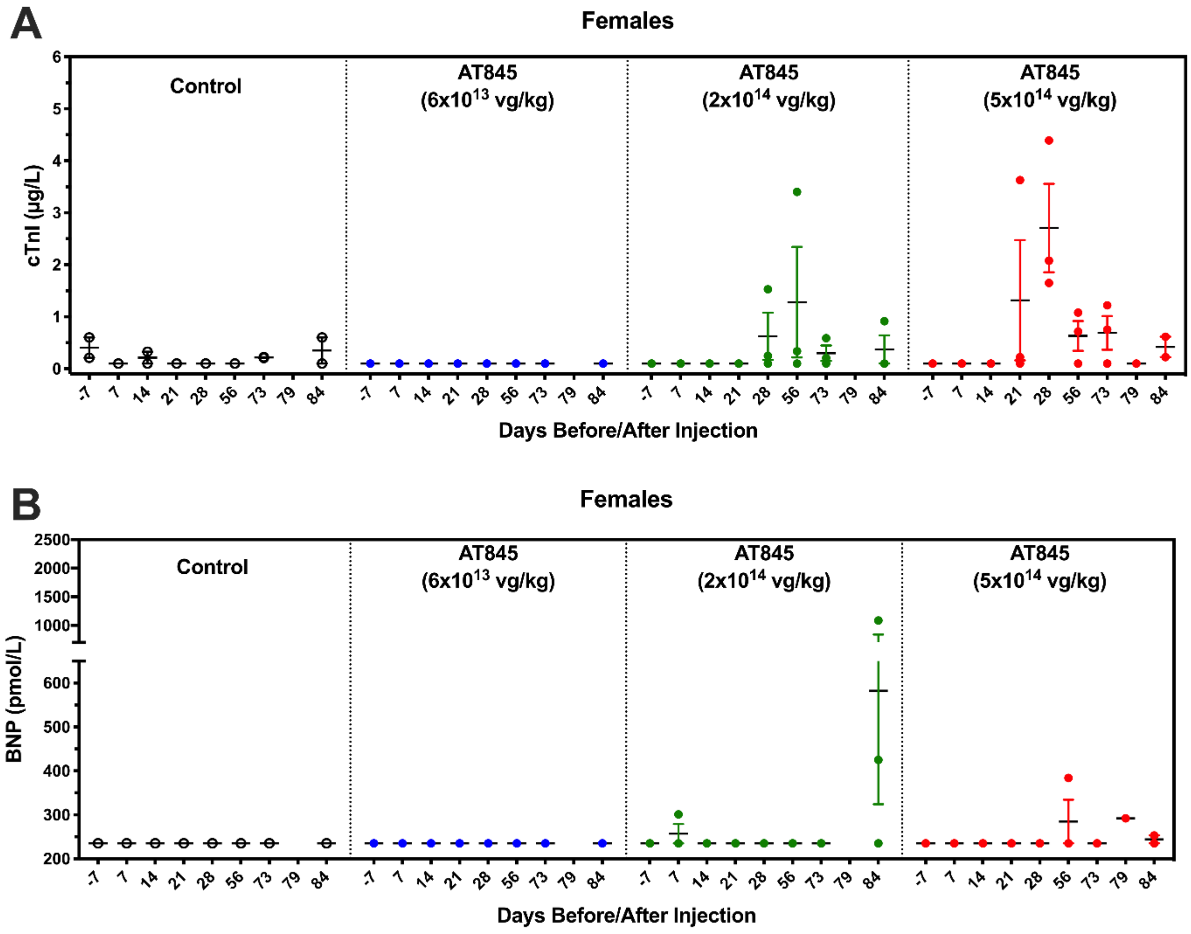

Appendix Figure S2. Cardiac biomarkers in female NHPs. Female cynomolgus monkeys were treated with vehicle control (N = 2) or AT845 (N = 3) at 6×10^{13}, 2×10^{14}, or 5×10^{14} vg/kg and followed for approximately 12 weeks. (A) Cardiac troponin I (cTnI) and (B) brain natriuretic peptide (BNP). Bars show mean and SEM.

Appendix Table S1. Glycogen levels in the muscle of wild-type and *Gaa*$^{-/-}$ mice.

Tissue	Genotype (Dose)	N	Mean*	SD	SEM	Lower 95%	Upper 95%
Heart	Wild-type (0)	8	0.98	2.26	0.80	(0.91)	2.87
	Gaa$^{-/-}$ (0)	8	925.87	590.65	208.83	432.08	1,419.67
	Gaa$^{-/-}$ (3×10^{13} vg/kg)	8	86.27	105.81	37.41	(2.19)	174.72
	Gaa$^{-/-}$ (1×10^{14} vg/kg)	8	2.77	3.71	1.31	(0.33)	5.87
	Gaa$^{-/-}$ (3×10^{14} vg/kg)	8	0.22	0.61	0.22	(0.29)	0.72
Quadriceps	Wild-type (0)	8	14.02	9.93	3.51	5.72	22.32
	Gaa$^{-/-}$ (0)	8	358.68	129.30	45.72	250.58	466.78
	Gaa$^{-/-}$ (3×10^{13} vg/kg)	8	220.31	223.74	79.10	33.26	407.36
	Gaa$^{-/-}$ (1×10^{14} vg/kg)	8	63.10	38.28	13.54	31.09	95.10
	Gaa$^{-/-}$ (3×10^{14} vg/kg)	8	18.40	9.86	3.49	10.15	26.64
Diaphragm	Wild-type (0)	8	206.69	221.26	78.23	21.71	391.66
	Gaa$^{-/-}$ (0)	8	792.48	320.46	113.30	524.57	1,060.39
	Gaa$^{-/-}$ (3×10^{13} vg/kg)	8	463.27	159.47	56.38	329.95	596.59
	Gaa$^{-/-}$ (1×10^{14} vg/kg)	8	90.66	75.06	26.54	27.90	153.42
	Gaa$^{-/-}$ (3×10^{14} vg/kg)	8	72.51	89.24	31.55	(2.09)	147.11

*Glycogen levels below LLOQ were assigned a value of zero (0) for this calculation.

Appendix Table S2. Echocardiographic findings in NHPs.

Group	Animal	Sex	Dose Group (vg/kg)	Echocardiographic Findings
1	1001	M	0	Mild tricuspid regurgitation
	1002	M	0	
	1501	F	0	Slight tricuspid regurgitation
	1502	F	0	
2	2001	M	6×10^{13}	Mild pulmonic regurgitation
	2002	M	6×10^{13}	Mild tricuspid regurgitation
	2003	M	6×10^{13}	Mild tricuspid regurgitation; Mild pulmonic regurgitation
	2501	F	6×10^{13}	
	2502	F	6×10^{13}	Mild tricuspid regurgitation
	2503	F	6×10^{13}	Mild tricuspid regurgitation
3	3001	M	2×10^{14}	Mild pulmonic regurgitation
	3002	M	2×10^{14}	Mild to moderate tricuspid regurgitation; Slight mitral regurgitation
	3003	M	2×10^{14}	
	3501	F	2×10^{14}	Mild tricuspid regurgitation; Mild pulmonic regurgitation
	3502	F	2×10^{14}	
	3503	F	2×10^{14}	Slight tricuspid regurgitation
4	4001	M	5×10^{14}	
	4002	M	5×10^{14}	
	4003	M	5×10^{14}	Slight tricuspid regurgitation; Mild pulmonic regurgitation
	4501	F	5×10^{14}	Severe tricuspid regurgitation; Moderate pulmonic regurgitation; Severe mitral regurgitation
	4502	F	5×10^{14}	
	4503	F	5×10^{14}	Mild tricuspid regurgitation

Individual animal echocardiographic (ECHO) findings on Day 84.

Appendix Table S3. Relative quantification of the percentage of each GAA Western blot band based on their signal intensity.

Description	Tissue	Relative Percentage Based on Signal Intensity			
		110kDa	95kDa	76kDa	70kDa
AT845	Heart	NA	NA	88.75	11.25
AT845	Heart	NA	NA	86.96	13.04
AT845	Heart	0.50	0.23	70.06	29.21
Control	Heart	NA	NA	77.07	22.93
AT845	Quadriceps	10.02	0.36	83.56	6.06
AT845	Quadriceps	2.24	0.69	90.64	6.43
AT845	Quadriceps	6.74	0.66	68.49	24.11
Control	Quadriceps	NA	NA	100	NA

Description	Tissue	Relative Percentage Based on Signal Intensity			
		110kDa	95kDa	76kDa	70kDa
Cyno-AT845	Heart	0.03	0.44	39.59	59.94
Cyno-AT845	Heart	0.03	0.73	37.48	61.76
Cyno-AT845	Heart	0.02	0.92	44.16	54.91
Cyno-AT845	Quadriceps	0.31	1.02	47.03	51.65
Cyno-AT845	Quadriceps	0.48	1.24	47.55	50.72
Cyno-AT845	Quadriceps	0.13	3.31	48.13	48.43
