# Supplementary material for: Muscle‐directed gene therapy corrects Pompe disease and uncovers species‐specific GAA immunogenicity
Source: EMBO Mol Med. 2021 Dec 1;14(1):e13968. doi: 10.15252/emmm.202113968 (PMC8749482; doi:10.15252/emmm.202113968)
Supplement: Supplementary file 4 [file EMMM-14-e13968-s002.pdf]

EMBO Molecular Medicine

Muscle-directed gene therapy corrects Pompe disease and uncovers species-specific GAA immunogenicity

Michelle Eggers, Charles Vannoy, Jianyong Huang, Pravinkumar Purushothaman, Jacqueline Brassard, Carlos Fonck, Hui Meng, Mariah Prom, Mike Lawlor, Justine Cunningham, Chanchal Sadhu, and Fulvio Mavilio
DOI: 10.15252/emmm.202113968

Corresponding authors: Michelle Eggers (meggers@audentestx.com) , Fulvio Mavilio (fulvio.mavilio@unimore.it)

Review Timeline:

Submission Date:	14th Jan 21
Editorial Decision:	5th Feb 21
Revision Received:	30th Jul 21
Editorial Decision:	23rd Aug 21
Revision Received:	4th Nov 21
Accepted:	9th Nov 21

Editor: Zeljko Durdevic

Transaction Report:

(Note: With the exception of the correction of typographical or spelling errors that could be a source of ambiguity, letters and reports are not edited. Depending on transfer agreements, referee reports obtained elsewhere may or may not be included in this compilation. Referee reports are anonymous unless the Referee chooses to sign their reports.)

5th Feb 2021

Dear Dr. Eggers,

Thank you for the submission of your manuscript to EMBO Molecular Medicine. We have now received feedback from the three reviewers who agreed to evaluate your manuscript. As you will see from the reports below, the referees acknowledge the interest of the study but also raise serious concerns that should be addressed in a major revision.

Addressing the reviewers' concerns in full will be necessary for further considering the manuscript in our journal, and acceptance of the manuscript will entail a second round of review. EMBO Molecular Medicine encourages a single round of revision only and therefore, acceptance or rejection of the manuscript will depend on the completeness of your responses included in the next, final version of the manuscript. For this reason, and to save you from any frustrations in the end, I would strongly advise against returning an incomplete revision.

We realize that the current situation is exceptional on the account of the COVID-19/SARS-CoV-2 pandemic. Therefore, please let us know if you need more than six months to revise the manuscript.

I look forward to receiving your revised manuscript.

Yours sincerely,

Zeljko Durdevic

Zeljko Durdevic
Editor
EMBO Molecular Medicine

***** Reviewer's comments *****

Referee #1 (Comments on Novelty/Model System for Author):

A detailed report about the statistical analysis limit is reported in the response to the author

Referee #1 (Remarks for Author):

The paper from Eggers and colleagues provides important insight into the development of gene therapy for Pompe disease from the bench to the bedside. It also shed light on the complexity of the development of a gene therapy approach targeting the muscle for this disease. The results they showed seem to support the hypothesis that the expression of a human protein is maybe toxic in non-human primates, in particular when the method of delivery involves muscle and heart targeting. Such a conclusion may be helpful in the interpretation of other NHP data obtained in the context of gene transfer with AAV vectors, however, the data reported in this paper presents several methodological issues that require an extensive revision and, in this reviewer opinion, preclude the publication of the paper in EMBO molecular medicine. I prepared a detailed list of the methodological issues I identified:

1. The death of two monkeys, one male, and one female in the protocol is only reported in the supplementary information. Since the toxicity is dose-dependent and the deaths were observed in the high-dose cohort, I found this an important piece of data that needs to be reported in the main text and in the abstract.
2. In the study 72 Gaa-/- mice, 36 males, and 36 females were treated with vehicle or AAV-GAA vector at three doses. Additionally, 18 Gaa+/+, 9 males and 9 females were vehicle-treated. In supplementary methods, it is described how 5 males

and 5 females of each group were used for histological analysis and the remaining 4 for biochemical analysis. In figure 1 the N indicated in the legend is 7-8 and the data showed are 3-8. The format used in the figures complicates the evaluation of the results and it is difficult to understand which data derives from males or females. Also please state clearly that there were no deaths all along with the protocol or provide the real number at the end of the protocol.
3. In figure 1 glycogen measured in the diaphragm of wild-type mice looks too high, higher than levels measured in knock-out animals in other tissues. This is something hardly found in this model. Please provide a reference about this finding, together with a description of the method used for glycogen quantification, including if the samples were normalized for basal glucose levels in tissues. A PAS staining to clarify glycogen accumulation in the diaphragm is required.
4. Line 212 and 235 make reference to supplemental material for dorsal root ganglia and liver alterations in NHPs, no data were provided.
5. Echocardiographic findings were reported in the figure but the data were not shown. Please include the data in the manuscript including quantitative analysis of the parameters described in the text.
6. Figure 5A, according to methods, shows anti-human GAA levels, and this did not exclude the formation of antibodies anti-Cyno GAA. Please be sure to make this evident and change the conclusions.
7. In Western blot of figure 7 please quantify the bands and evaluate the ratio of the two isoforms (described as 76 and 70) in all the monkey of the study, including untreated animals. Please evaluate differences in processing between untreated animals and animals injected with AAV vectors expressing the two GAA protein (Human and Cyno). This referee is also not sure of the interpretation of the molecular weight of the bands, the highest band of the two seems more consistent to a 90 KDa molecular weight than 76. The 76 and 70 KDa bands should be more similar in electrophoretic migration, as seen in the heart of the untreated monkey in panel A.
8. Lines 296 and 303 in the discussion, the author claim that the levels of expression of GAA were never reached in other studies, in particular those involving liver gene transfer. However, in those studies, 10-fold lower AAV doses were used with similar efficacy in terms of glycogen clearance in the mouse model (i.e. complete glycogen clearance) and no adverse events with the human protein in NHPs. Please adapt the discussion to reflect this evidence.
9. Line 331 of the discussion. No proof of the incomplete processing of GAA in mice was provided and NHP data need quantification and comparison with untreated animals.
10. It is surprising to this reviewer that the toxicity of high doses of AAV vectors was not discussed particularly in light of the liver alterations observed in NHPs (line 236, data not shown and to be showed) and the recent reports of the death of three children in a clinical trial performed using the same AAV vector serotype.
11. Statistical analysis: statistical analyses were performed by two-way ANOVA. Could you please provide the two variables used for the analysis? Also in several cases, the statement in the text was not supported by statistical analysis. Some example:
a. Line 127, statistics in quadriceps do not support the statement, same for lines 130 and 132
b. Line 163 to 172, no stats performed, most of the conclusion are too strong
c. Line 216 to 227 many statements with no data shown including data from female missing

Referee #2 (Comments on Novelty/Model System for Author):

This is an important study that addresses unexpected toxicity related to an AAV vector developed to treat Pompe disease. The authors identified the basis for toxicity in the form of a xenogeneic immune response. Understanding the toxicity from a standard AAV vector intended for clinical development is important to the field. The study satisfied the standards for ethical use of the model organisms.

Referee #2 (Remarks for Author):

This is an important study that addresses unexpected toxicity related to an AAV vector developed to treat Pompe disease. The authors identified the basis for toxicity in the form of a xenogeneic immune response. There are only a few issues that should be addressed, listed below.
Major concerns:
1) At least two passages state that AT845 was well-tolerated or would be expected to be well-tolerated, when clearly the data demonstrated toxicity in NHPs:
a. Page 5, lines 98 to 100, and
b. Page 16, lines 327 to 328.
This study in no way demonstrates or supports safety of AT845 in NHPs or predicts safety in humans. All of the data for AT845 demonstrates toxicity from expressing human GAA in NHPs. There is no evidence that it is safe in NHPs, nor that it could be safely administered to CRIM-positive patients, some of whom mount immune responses against GAA. It is just as likely that muscle expression of human GAA with AT845 would provoke immunity in a clinical trial, and therefore these statements about safety are unsupported and cannot be allowed.
2) Inaccurate statements about liver-based gene therapy for Pompe disease, which should be corrected to avoid confusion:
a. Page 13, line 272 to 273: The statement that a "secretable GAA-encoding transgene" is needed for liver gene therapy. Han et al 2017 used a wild-type GAA gene, as did Sun et al 2007 and Zhang et al 2012. It would be more accurate to state that Han et al 2017 used a wildtype GAA transgene, and that Puzzo et al 2017 used a modified "secretable GAA".

b. Page 14, lines 296 to 299 state that liver-based gene therapy could not achieve "supraphysiological GAA activity [that] was required for rapid clearance of glycogen storages in the mouse model". This statement is inaccurate, because Puzzo et al 2017 and other studies with a liver-based strategy decreased muscle glycogen content by >90% in the skeletal muscle of GAA-KO mice to levels indistinguishable from wildtype levels.
3) Need for additional statistical analysis to demonstrate scientific rigor of this study. Please provide statistical analysis and show P values for Figure 1, showing differences for treated groups both from wild-type and GAA-KO control mice. Using two different symbols can accomplish this readily. Similarly this is needed for Figure 3, especially to support the claim in lines 172 to 173. P values should be shown in Figure 4, Figure 5, Figure 7 (add a graph showing quantification of the immunoblotting data), and in Figure S3.
4) The main pathology has been relegated to Supplemental Results in Figures S4 and S5, and warrants publication in the main article. Hi-magnification inserts showing necrotic cells should be provided.
Minor concerns
1) Lines 150 and 236 cite Supplemental Results without specifying which figure or table is referenced.
2) Line 323 referring to the sequence of macaque and human GAA needs a reference.
3) The Methods need to include the method for glycogen content.
4) Line 41 has a typographical error, "hon-human".

Referee #3 (Remarks for Author):

The study describes the effects of systemic administration of AAV8-hGAA (AT845).
- In mice, all 3 doses showed increase in GAA protein, GAA activity, and glycogen clearance in heart and quadriceps muscles.
- In mice, all 3 doses showed normal body weight growth overtime.
- NHP were also been tested with similar results.

1. The involvement of body weight in GT-treated especially in lower and mid dose group but only transient in Gaa-/- mice may have some clinical impact. Further explanation/ observation will be needed. (Fig 3A, vehicle treated?)
2. The improvement of grip test in some but not all animals need further correlation. Suggest to add the percentage of failure or success in each group for better understanding. How about the reasons for those without improvement, more residual glycogen?
3. As for the supraphysiology levels of GAA expression, how about the systemic excretion GAA activity? Why dose the liver only have the background GAA activity?
4. Since AT845 only express in muscle and heart, what's the reason for the observation of inflammation in both dorsal root ganglia and nerves? Any GAA expression there? Or is AAV8 the antigen? No data of empty AAV8 vector at mid and high dose?

POINT-BY-POINT REPLY

Referee #1

1. *The death of two monkeys, one male, and one female in the protocol is only reported in the supplementary information (…) I found this an important piece of data that needs to be reported in the main text and in the abstract.*

The death of the two monkeys is now reported in the Results section of the main text (p. 11, lines 231-233) and abstract (p. 2, lines 36-37).

2. *In the study 72 Gaa-/- mice, 36 males, and 36 females were treated with vehicle orAAV-GAA vector at three doses (…). In figure 1 the N indicated in the legend is 7-8 and the data showed are 3-8. The format used in the figures complicates the evaluation of the results and it is difficult to understand which data derives from males or females. Also please state clearly that there were no deaths all along with the protocol or provide the real number at the end of the protocol.*

The number of surviving animals in each cohort at the end of the study is now indicated in the Appendix Supplemental Results, and the number of animals tested for each parameter (VCN, GAA activity, GAA levels, glycogen levels) is indicated in the Figure 1 legend. The number of tissue samples analyzed varied across treatment groups and assays because some mice were too small to yield sufficient tissue for every assay.

Distinguishing all figures by male and female mice would make the figures difficult to read because the data points for both sexes are clustered together. As the one endpoint in which there was a clear separation between males and females, the grip strength response test is now graphed distinguishing males from females. Additional analyses of VCN, GAA, and glycogen levels did not help explain why females, compared to males, performed better in the grip response test at the low and mid doses. This statement was added to the manuscript in Results (p. 9, lines 183-185).

3. *In figure 1 glycogen measured in the diaphragm of wild-type mice looks too high (…). Please provide a reference about this finding, together with a description of the method used for glycogen quantification, including if the samples were normalized for basal glucose levels in tissues. A PAS staining to clarify glycogen accumulation in the diaphragm is required.*

The method used for glycogen quantification is now described in the Materials and Methods. We have used a glycogen quantification kit that selectively detects muscle glycogen through a stepwise in vitro glucoamylase hydrolysis reaction. Also, we did not normalize the values for glucose content in the muscle samples because skeletal muscles are typically unable to release glucose from stored muscle glycogen and it is mostly broken down to lactate in the muscle. The diaphragm tissue was heated and denatured for glycogen staining, thus PAS staining was not possible.

Glycogen levels are inherently higher in diaphragm compared with heart or skeletal muscle in the GAA$^{-/-}$ mouse model (Keeler *et al.* 2020; Han *et al.* 2019). This information is now referenced in the Conclusions section (p. 15, lines 325-328). In addition, it has been reported that glycogen levels can have daily fluctuations up to +/- 100% (Gomes *et al*. 2009), which in part can explain the variability observed in the data obtained.

4. *Line 212 and 235 make reference to supplemental material for dorsal root ganglia and liver alterations in NHPs, no data were provided.*

Micrographic images from liver and DRG sections are now included in Figures EV5 and EV6, respectively.

5. *Echocardiographic findings were reported in the figure but the data were not shown. Please include the data in the manuscript including quantitative analysis of the parameters described in the text.*

Echocardiographic assessments were qualitative only. A new Appendix Table S2 lists notable echocardiographic findings for individual monkeys.

6. *Figure 5A, according to methods, shows anti-human GAA levels, and this did not exclude the formation of antibodies anti-Cyno GAA. Please be sure to make this evident and change the conclusions.*

The anti-GAA total antibody assay we used did not distinguish between anti-human and anti-Cyno GAA antibodies. Monkeys were dosed with a construct specifically expressing recombinant human GAA. Therefore, the anti-GAA antibodies detected are likely reacting against the heterologous human GAA protein. It is also possible that anti-human-GAA antibodies could cross-react with Cyno-GAA, resulting in a brake of tolerance. In effect, anti-GAA antibodies were not detected in cynomolgus monkeys dosed with a Cyno-specific construct that resulted in the expression of high levels of Cyno GAA protein, which further supports the hypothesis that the Cyno GAA construct did not raise a humoral immune response and anti-GAA antibodies detected in the monkeys are due to cross-reactivity targeting the GAA protein from both species. We have noted in the manuscript that we cannot distinguish between the two types of antibodies in the Results (p. 11, lines 227-231), and adjusted the Figure 5 legend accordingly.

7. *In Western blot of figure 7 please quantify the bands and evaluate the ratio of the two isoforms (described as 76 and 70) in all the monkey of the study, including untreated animals. Please evaluate differences in processing between untreated animals and animals injected with AAV vectors expressing the two GAA protein (Human and Cyno).*

Post-transcriptional processing of the GAA protein was evaluated in vehicle-treated cynomolgus monkeys as well as monkeys dosed with either human GAA or Cyno GAA constructs. A ratio of

the processed GAA bands (76 and 70 kDa bands) was calculated based on their mean intensities observed on the Western blot. A new Appendix Table S3 provides the relative intensities of each band. The differences in banding patterns observed for the vehicle-treated animals is likely attributable to the lower basal level expression of GAA.

8. *Lines 296 and 303 in the discussion, the author claim that the levels of expression of GAA were never reached in other studies, in particular those involving liver gene transfer. However, in those studies, 10-fold lower AAV doses were used with similar efficacy in terms of glycogen clearance in the mouse model (i.e. complete glycogen clearance) and no adverse events with the human protein in NHPs. Please adapt the discussion to reflect this evidence.*

This referee is correct in noting that the AAV doses used (~ 10-fold lower) in liver gene transfer studies led to glycogen clearance in the Pompe mouse model with no apparent toxicity in NHPs. However, the vector biodistribution, and thus the GAA uptake in mice is not comparable with that of monkeys (and expected in patients), where the liver-targeting dose could be insufficient to restore GAA homeostasis. In fact, the GAA activity reported by Puzzo *et al*. (2017) in NHPs dosed with a liver-directed AAV at a dose of $2x10^{12}$ vg/kg was barely higher than in control animals in skeletal muscle, and about 3-fold higher in heart and diaphragm. Those levels could be insufficient to clear glycogen accumulation and restore homeostasis in patients. We believe that the GAA levels obtained in muscle by our approach, albeit at a higher dose, are much more robust and thus more likely to achieve therapeutic benefit in severe Pompe patients. Nevertheless, as requested by this reviewer, we have amended the discussion to explicitly note that liver-directed gene therapy requires a lower and potentially more tolerable dose than that required by a muscle-directed approach (Discussion, p. 16, lines 324-327)

9. *Line 331 of the discussion. No proof of the incomplete processing of GAA in mice was provided (….)*

We have modified the discussion to reflect that inefficient processing of human GAA in mice is hypothetical (Discussion, p. 17, lines 364-367).

10. *It is surprising to this reviewer that the toxicity of high doses of AAV vectors was not discussed particularly in light of the liver alterations observed in NHPs (line 236, data not shown and to be showed) and the recent reports of the death of three children in a clinical trial performed using the same AAV vector serotype.*

Test article-related changes in the liver of NHPs in this study consisted of transient elevations of liver enzymes and observations of mononuclear cell infiltrates. These liver effects are now shown in Figure EV5 and described in the figure legend. We indicate that these liver findings are immune-mediated because they only occurred in monkeys that received the human version of the GAA coding sequence, and not in monkeys that received the Cyno version of the transgene. Taken together, these results are consistent with a xenogeneic response against the

heterologous human protein, not the AAV8 capsid nor GAA expression *per se*, and thus are unlikely to be observed in Pompe patients receiving AT845.

The reviewer's comment is in reference to the fatalities observed in the ASPIRO trial, a Phase 1/2 clinical trial with XLMTM patients <5 years of age that received AT132 for the de novo expression of myotubularin in muscle tissue. As previously presented (Shieh et al, 2020; Bonneman et al ASGCT 2021), SAEs observed in the ASPIRO trial are likely the result of AAV-mediated exacerbation of pre-existing hyperbilirubinemia (Molera *et al.* 2021) leading to uncompensated liver disease in XLMTM patients (Shieh *et al.* 2020). As an aside, there were no observations of hyperbilirubinemia or uncompensated liver disease in the pre-clinical studies performed with AT132 (Shieh *et al.* 2020). AT845 is an AAV gene replacement therapy for late-onset Pompe disease in patients ≥18 years of age. There are no data, from this or any other studies, that point to shared outcomes or mechanisms between AT845 and AT132 in terms of liver effects. We anticipate, that in time, additional data will be shared highlighting potential mechanisms underlying the SAEs in the ASPIRO trial, which would be beyond the scope of this manuscript.

11. *Statistical analysis: statistical analyses were performed by two-way ANOVA. Could you please provide the two variables used for the analysis?*

Statistical analyses were performed by two-way ANOVA followed by Dunnett's multiple comparisons test. The dependent variables are GAA activity or glycogen concentration. The independent variables are mouse genotype and dose.

Also in several cases, the statement in the text was not supported by statistical analysis. Some example:

a. Line 127, statistics in quadriceps do not support the statement, same for lines 130 and 132.

Information on the statistical test used and the level of significance in the comparisons between treated and untreated GAA KO mice are now provided in the legend of Figure 1. The text summarizes the results, we didn't systematically report statistical significances dose by dose and tissue by tissue for GAA activity and glycogen levels as they are clearly presented in the figure.

b. Line 163 to 172, no stats performed, most of the conclusion are too strong

Results of statistical analyses were added to Figure 3. We removed the word significantly in the text when describing the difference in response between males and females since differences between males and females were not formally tested (p. 9, line 181).

c. Line 216 to 227 many statements with no data shown including data from female missing

Figure 5 shows male only data, as the study conducted with the Cyno GAA construct was only completed in males. This allows the viewer to compare directly between studies for the same gender. Appendix Figure S2 shows Cardiac troponin I (cTnI) and (B) brain natriuretic peptide in the females. Appendix Table S2 was added to support statements regarding the echocardiogram findings in both males and females.

Referee #2

We thank this reviewer for his/her appreciation of the importance of our study.

Major concerns:

1. *At least two passages state that AT845 was well-tolerated or would be expected to be well-tolerated, when clearly the data demonstrated toxicity in NHPs: a. Page 5, lines 98 to 100, and b. Page 16, lines 327 to 328.*

In agreement with the reviewer's comment, we now state that AT845 was well-tolerated when the species-specific transgene is used (Introduction, p. 5, lines 100-101). The sentence on page 16 was edited to suggest that the current data may predict safety of expressing human GAA in CRIM-positive patients (Discussion, p. 17, line 386). Since the vast majority of CRIM-positive patients are tolerant to enzyme replacement therapy (ERT), and do not develop anti-GAA antibodies, we believe it is reasonable to expect that synthesis of the human enzyme in the muscle tissue of patients will be safe. The Phase 1 clinical study is designed to directly address this question.

This study in no way demonstrates or supports safety of AT845 in NHPs or predicts safety in humans. All of the data for AT845 demonstrates toxicity from expressing human GAA in NHPs. There is no evidence that it is safe in NHPs, nor that it could be safely administered to CRIM-positive patients, some of whom mount immune responses against GAA. It is just as likely that muscle expression of human GAA with AT845 would provoke immunity in a clinical trial, and therefore these statements about safety are unsupported and cannot be allowed.

The data generated in this study support the claim that AT845 is safe in non-human primates at doses as high as $2x10^{14}$ vg/kg provided that the homologous gene is expressed. As discussed in the manuscript, the explanation that the heterologous expression of the human gene in NHP can cause a xenogeneic immune response resulting in the adverse events observed is, in our view, perfectly plausible. We stress the point that expression of a human protein may cause toxicity in primate tox studies more frequently than previously suspected, and thus caution should be used before concluding that an AAV product is toxic in the presence of humoral or cell-mediated immune responses against heterologous transgene proteins.

The FDA guidelines allow the use of a species-specific transgene in toxicology studies when there is an appropriate scientific justification: *"However, in certain cases, due to the species-specific nature of the clinical product (e.g., some vector-expressed human transgenes: human-derived cellular therapy (CT) products), testing the CGT product intended for clinical administration in animals may not be informative, and therefore testing of an analogous product may be a suitable alternative."* (FDA Guidance to Industry: Preclinical assessment of Investigational Cellular and Gene Therapy Products, November 2013, https://www.fda.gov/regulatory-information/search-fda-guidance-documents/preclinical-

assessment-investigational-cellular-and-gene-therapy-products). The adverse findings in this study are considered and discussed in the manuscript in the context of a potential xenogeneic immune response. Further, a central aim of this study was to test this very hypothesis by generating a human and a cynomolgus version of the GAA transgene and comparing their effects. We note that the FDA cleared our AT845 Phase 1/2 clinical trial, and patients have been dosed in the trial.

2. *Inaccurate statements about liver-based gene therapy for Pompe disease, which should be corrected to avoid confusion:*

a. Page 13, line 272 to 273: The statement that a "secretable GAA-encoding transgene" is needed for liver gene therapy. Han et al 2017 used a wild-type GAA gene, as did Sun et al 2007 and Zhang et al 2012. It would be more accurate to state that Han et al 2017 used a wildtype GAA transgene, and that Puzzo et al 2017 used a modified "secretable GAA".

In agreement with the reviewer, we changed the sentence (Discussion, p. 14, lines 293-294).

b. Page 14, lines 296 to 299 state that liver-based gene therapy could not achieve "supraphysiological GAA activity [that] was required for rapid clearance of glycogen storages in the mouse model". This statement is inaccurate, because Puzzo et al 2017 and other studies with a liver-based strategy decreased muscle glycogen content by >90% in the skeletal muscle of GAA-KO mice to levels indistinguishable from wildtype levels.

In agreement with the reviewer, we changed the sentence in the Discussions section (Discussion, p. 15, lines 324-327).

3. *Need for additional statistical analysis to demonstrate scientific rigor of this study. Please provide statistical analysis and show P values for Figure 1, showing differences for treated groups both from wild-type and GAA-KO control mice. Using two different symbols can accomplish this readily. Similarly this is needed for Figure 3, especially to support the claim in lines 172 to 173. P values should be shown in Figure 4, Figure 5, Figure 7 (add a graph showing quantification of the immunoblotting data), and in Figure S3.*

Figures 1, 3, 4, and S1 (previously Figure S2), and EV2 (previously Figure S3) have been changed according to the reviewer's suggestions.

We removed the word significantly in the text (previously lines 172 and 173) when describing the difference in response between males and females shown in Figure 3, since differences between males and females were not statistically analyzed.

Statistical analyses were not conducted for data shown in Figure 5 because of the low n's in each group (n = 2-3).

A new Appendix Table S3 provides relative quantification of each protein band based on signal intensity.

4. *The main pathology has been relegated to Supplemental Results in Figures S4 and S5, and warrants publication in the main article. Hi-magnification inserts showing necrotic cells should be provided.*

The former Figures S4 and S5 (now Figures EV3 and EV4) have been enlarged to meet the other reviewers' request. We also added high-magnification inserts of the necrotic cells, as suggested by this reviewer. Placement of these images in the supplementary section is consistent with the Journal's guidelines.

Minor concerns

1) *Lines 150 and 236 cite Supplemental Results without specifying which figure or table is referenced.*

The citation refers to a specific section of text in the former Supplemental files (now Appendix) called Supplemental Results. The Supplemental Results integrate the description of the pathology findings in both mice and NHPs.

2) *Line 323 referring to the sequence of macaque and human GAA needs a reference*.

References to the relevant UniProt identifiers have been added in the text.

3) *The Methods need to include the method for glycogen content.*

The glycogen method has been added to the Methods section.

4) *Line 41 has a typographical error, "hon-human".*

Thank you, the typo is now corrected.

Referee #3

1. *The involvement of body weight in GT-treated especially in lower and mid dose group but only transient in Gaa-/- mice may have some clinical impact. Further explanation/observation will be needed. (Fig 3A, vehicle treated?)*

An explanation was added to the Results section (p. 8, lines 160-165).

2. *The improvement of grip test in some but not all animals need further correlation. Suggest to add the percentage of failure or success in each group for better understanding. How about the reasons for those without improvement, more residual glycogen?*

The percentage of failure for each group has been added in Figure 3 legend. This reviewer is probably correct in that some animals may have responded less to the gene therapy, possibly as a consequence of lower transduction in the limb muscles. Unfortunately, vector copy number (VCN) was not measured in the animals that underwent functional testing.

3. *As for the supraphysiology levels of GAA expression, how about the systemic excretion GAA activity? Why dose the liver only have the background GAA activity?*

We do not express significant secretion of GAA in these animals. In AT845, the GAA cDNA is under the control of the MCK enhancer/promoter combination, which restricts expression to muscle. Neither skeletal muscle, nor the heart are efficient at secreting proteins into the bloodstream. On the other hand, expression in the liver is minimal, since the promoter showed a very low-level of activity in hepatocytes, as shown by RNA, protein and enzymatic activity analysis.

4. *Since AT845 only express in muscle and heart, what's the reason for the observation of inflammation in both dorsal root ganglia and nerves? Any GAA expression there? Or is AAV8 the antigen? No data of empty AAV8 vector at mid and high dose?*

In our studies, the DRG changes consisted of neuronal degeneration and satellite cell (glial cell) hypertrophy/hyperplasia (or gliosis) more so than inflammation. Similar changes have been reported in published NHP studies with systemic administration of AAV8 or AAV9 vectors. The DRG change is a source of controversy, as some investigators believe that transgene expression is at least a concomitant cause (e.g., Hordeaux *et al*. 2020). We suspect that DRG neurons are sensitive to AAV capsids (Discussion, p. 16, lines 339-345). Unfortunately, we did not inject NHPs with empty capsid controls to formally support this hypothesis.

References

Gomes FR, Rezende EL, Malisch JL, et al. Glycogen storage and muscle glucose transporters (GLUT-4) of mice selectively bred for high voluntary wheel running. J Exp Biol. 2009;212:238-248.

Han SO, Li S, McCall A, et al. Comparisons of infant and adult mice reveal age effects for liver depot gene therapy in Pompe disease. Mol Ther Methods Clin Dev. 2020;17:133-142.

Hordeaux J, Buza EL, Jeffrey B, et al. MicroRNA-mediated inhibition of transgene expression reduces dorsal root ganglion toxicity by AAV vectors in primates. Sci Transl Med. 2020 Nov 11;12(569):eaba9188.

Keeler AM, Xieger M, Todeasa SH, et al. Systemic delivery of AAVB1-GAA clears glycogen and prolongs survival in a mouse model of Pompe disease. Hum Gene Ther. 2019;30:57-68.

Molera C, Sarishvili T, Nascimento A, et al. Intrahepatic cholestasis is a clinically significant feature associated with natural history of X-linked myotubular myopathy (XLMTM): a case series and biopsy report. J Neuromusc Dis. 2021; Accepted for publication.

Puzzo F, Colella P, Biferi MG, et al. Rescue of Pompe disease in mice by AAV-mediated liver delivery of secretable acid α-glucosidase. Sci Transl Med. 2017;9(418):eaam6375.

Shieh PB, Bönnemann CG, Müller-Felber W, et al. Re: "Moving Forward After Two Deaths in a Gene Therapy Trial of Myotubular Myopathy" by Wilson and Flotte. Hum Gene Ther. 2020; 31(15-16): 787.

1st Revision - Editorial Decision **23rd Aug 2021**

23rd Aug 2021

Dear Dr. Eggers,

Thank you for the submission of your revised manuscript to EMBO Molecular Medicine. We have now heard back from the three referees who we asked to re-evaluate your manuscript. As you will see from the reports below, the referees are overall supporting publication of your manuscript. However, referee #1 raises an important concern regarding possibly missing/excluded data points and statistical analysis. Please address these points and provide raw data for the experiments with mice in an additional and final round of revision.

Acceptance or rejection of the manuscript will depend on the completeness of your responses included in the next, final version of the manuscript and will entail an additional round of review. For this reason, and to save you from any frustrations in the end, I would strongly advise against returning an incomplete revision.

In addition, please amend the following:
1) In the main manuscript file, please do the following:
- Correct/answer the track changes suggested by our data editors by working from the uploaded document.
- Remove "data not shown" (p.11).
- Remove all figures from the main manuscript file and leave figure legends.
- Add callouts for EV Fig 3-6 and Appendix Table S2.
- In M&M, provide the antibody dilutions that were used for each antibody.
- In M&M, the statistical paragraph should reflect all information that you have filled in the Authors Checklist, especially regarding randomization, blinding, replication.
2) Appendix: Please move supplemental results to the main manuscript text and rename figure legends and tables to "Appendix Figure S1" and "Appendix Table S1" etc. Also, remove separately uploaded appendix figures.
3) The Paper Explained: Please provide "The Paper Explained" and add it to the main manuscript text. Please check "Author Guidelines" for more information. https://www.embopress.org/page/journal/17574684/authorguide#researcharticleguide
4) Synopsis:
- Synopsis image: Please provide the synopsis image as a 550 px-wide x (250-400)-px high high-resolution jpeg file.
- Please check your synopsis text and image, revise them if necessary and submit their final versions with your revised manuscript. Particular attention should be given to grammar and syntax. Please be aware that in the proof stage minor corrections only are allowed (e.g., typos).
5) Source data: We encourage you to include the source data for figure panels that show essential data. Numerical data should be provided as individual .xls or .csv files (including a tab describing the data). For blots or microscopy, uncropped images should be submitted (using a zip archive if multiple images need to be supplied for one panel). Please check "Author Guidelines" for more information. https://www.embopress.org/page/journal/17574684/authorguide#sourcedata
6) For more information: There is space at the end of each article to list relevant web links for further consultation by our readers. Could you identify some relevant ones and provide such information as well? Some examples are patient associations, relevant databases, OMIM/proteins/genes links, author's websites, etc...
7) As part of the EMBO Publications transparent editorial process initiative (see our Editorial at http://embomolmed.embopress.org/content/2/9/329), EMBO Molecular Medicine will publish online a Review Process File (RPF) to accompany accepted manuscripts. This file will be published in conjunction with your paper and will include the anonymous referee reports, your point-by-point response and all pertinent correspondence relating to the manuscript. Let us know whether you agree with the publication of the RPF and as here, if you want to remove or not any figures from it prior to publication. Please note that the Authors checklist will be published at the end of the RPF.
8) Please provide a point-by-point letter INCLUDING my comments as well as the reviewer's reports and your detailed responses (as Word file).

I look forward to seeing a revised form of your manuscript as soon as possible. Use this link to login to the manuscript system and submit your revision: https://embomolmed.msubmit.net/cgi-bin/main.plex

Yours sincerely,

Zeljko Durdevic

Zeljko Durdevic
Editor
EMBO Molecular Medicine

***** Reviewer's comments *****

Referee #1 (Comments on Novelty/Model System for Author):

There are missing data points in the paper and I cannot accept as an explanation the sentence: ' The number of tissue samples analyzed varied across treatment groups and assays because some mice were too small to yield sufficient tissue for every assay'. We routinely perform these assays and they do not require much material. So if there are excluded values or missing data points they should be clearly indicated. Also, I have some doubts about the statistical analyses. In table S1 the authors report the data of glycogen measured in mice and in this table the reported number of mice is N=8.

Referee #1 (Remarks for Author):

The authors addressed most of my concerns. However, one fundamental issue remains in the paper related to the mouse data. There are missing data points and I cannot accept as an explanation the sentence: ' The number of tissue samples analyzed varied across treatment groups and assays because some mice were too small to yield sufficient tissue for every assay'. Also, in table S1 the authors report the data of glycogen measured in mice and in this table the reported number of mice is N=8. Based on this I am asking the authors to include tables with raw data (including GAA activity, glycogen, and mouse or tissue weight) with the exact mouse number and thorough statistical analysis.

Referee #2 (Comments on Novelty/Model System for Author):

This is a preclinical study of toxicity from AAV vector gene therapy using standard methods. While technical quality is high, novelty and medical impact are average based upon the topic and prior work.

Referee #2 (Remarks for Author):

All of my concerns were addressed in the revised manuscript and responses to reviewers.

Referee #3 (Remarks for Author):

Is suitable for publication

Referee #1

1. *The authors addressed most of my concerns. However, one fundamental issue remains in the paper related to the mouse data. There are missing data points and I cannot accept as an explanation the sentence: ' The number of tissue samples analyzed varied across treatment groups and assays because some mice were too small to yield sufficient tissue for every assay'. Also, in table S1 the authors report the data of glycogen measured in mice and in this table the reported number of mice is N=8. Based on this I am asking the authors to include tables with raw data (including GAA activity, glycogen, and mouse or tissue weight) with the exact mouse number and thorough statistical analysis.*

The raw data for the GAA activity, glycogen, and mouse body weight is included with this resubmission. The glycogen analysis yielded results from n=8 per group; however, the vector copy number analysis yielded n=7 per group for the two control groups and n=8 for the remaining dosing groups in heart tissue. The VCN yielded n=7 per control group because there was not sufficient tissue for this assay as well as the other assays conducted on the same heart tissue, due to the small size of the mice. Tissue weights taken for each assay were not recorded.

Referee #2

1. *All of my concerns were addressed in the revised manuscript and responses to reviewers..*

Thank you.

Referee #3

1. *Is suitable for publication.*

Thank you.

We are pleased to inform you that your manuscript is accepted for publication and is now being sent to our publisher to be included in the next available issue of EMBO Molecular Medicine.

EMBO PRESS

YOU MUST COMPLETE ALL CELLS WITH A PINK BACKGROUND ↓

PLEASE NOTE THAT THIS CHECKLIST WILL BE PUBLISHED ALONGSIDE YOUR PAPER

Corresponding Author Name:	Michelle Eggers
Journal Submitted to:	EMBO Molecular Medicine
Manuscript Number:	EMM-2021-13968

Reporting Checklist For Life Sciences Articles (Rev. June 2017)

This checklist is used to ensure good reporting standards and to improve the reproducibility of published results. These guidelines are consistent with the Principles and Guidelines for Reporting Preclinical Research issued by the NIH in 2014. Please follow the journal's authorship guidelines in preparing your manuscript.

A- Figures
1. Data
The data shown in figures should satisfy the following conditions:

- ➔ the data were obtained and processed according to the field's best practice and are presented to reflect the results of the experiments in an accurate and unbiased manner.
- ➔ figure panels include only data points, measurements or observations that can be compared to each other in a scientifically meaningful way.
- ➔ graphs include clearly labeled error bars for independent experiments and sample sizes. Unless justified, error bars should not be shown for technical replicates.
- ➔ if n< 5, the individual data points from each experiment should be plotted and any statistical test employed should be justified
- ➔ Source Data should be included to report the data underlying graphs. Please follow the guidelines set out in the author ship guidelines on Data Presentation.

2. Captions

Each figure caption should contain the following information, for each panel where they are relevant:

- ➔ a specification of the experimental system investigated (eg cell line, species name).
- ➔ the assay(s) and method(s) used to carry out the reported observations and measurements
- ➔ an explicit mention of the biological and chemical entity(ies) that are being measured.
- ➔ an explicit mention of the biological and chemical entity(ies) that are altered/varied/perturbed in a controlled manner.

- ➔ the exact sample size (n) for each experimental group/condition, given as a number, not a range;
- ➔ a description of the sample collection allowing the reader to understand whether the samples represent technical or biological replicates (including how many animals, litters, cultures, etc.).
- ➔ a statement of how many times the experiment shown was independently replicated in the laboratory.
- ➔ definitions of statistical methods and measures:
  - common tests, such as t-test (please specify whether paired vs. unpaired), simple χ2 tests, Wilcoxon and Mann-Whitney tests, can be unambiguously identified by name only, but more complex techniques should be described in the methods section;
  - are tests one-sided or two-sided?
  - are there adjustments for multiple comparisons?
  - exact statistical test results, e.g., P values = x but not P values < x;
  - definition of 'center values' as median or average;
  - definition of error bars as s.d. or s.e.m.

Any descriptions too long for the figure legend should be included in the methods section and/or with the source data.

USEFUL LINKS FOR COMPLETING THIS FORM

http://www.antibodypedia.com
http://1degreebio.org
http://www.equator-network.org/reporting-guidelines/improving-bioscience-research-repor
http://grants.nih.gov/grants/olaw/olaw.htm
http://www.mrc.ac.uk/Ourresearch/Ethicsresearchguidance/Useofanimals/index.htm
http://ClinicalTrials.gov
http://www.consort-statement.org
http://www.consort-statement.org/checklists/view/32-consort/66-title
http://www.equator-network.org/reporting-guidelines/reporting-recommendations-for-tumo
http://datadryad.org
http://figshare.com
http://www.ncbi.nlm.nih.gov/gap
http://www.ebi.ac.uk/ega
http://biomodels.net/
http://biomodels.net/miriam/
http://jjj.biochem.sun.ac.za
http://oba.od.nih.gov/biosecurity/biosecurity_documents.html
http://www.selectagents.gov/

In the pink boxes below, please ensure that the answers to the following questions are reported in the manuscript itself. Every question should be answered. If the question is not relevant to your research, please write NA (non applicable). We encourage you to include a specific subsection in the methods section for statistics, reagents, animal models and human subjects.

B- Statistics and general methods

Please fill out these boxes ↓ (Do not worry if you cannot see all your text once you press return)

1.a. How was the sample size chosen to ensure adequate power to detect a pre-specified effect size?	The total number of animals used, as well as the group size and number of groups, in this study was considered to be the minimum required to properly characterize the effects of AT845 as required to support regulatory submissions and was designed such that it does not require an unnecessary number of animals to accomplish its objectives.
1.b. For animal studies, include a statement about sample size estimate even if no statistical methods were used.	The sample size chosen was based on the study design guidelines specific for preclinical studies for gene replcement therapies. (1) ICH Harmonised Tripartite Guideline M3 (R2). Nonclinical Safety Studies for the Conduct of Human Clinical Trials and Marketing Authorization for Pharmaceuticals. (2) Guidance for Industry Gene Therapy Clinical Trials – Observing Subjects for Delayed Adverse Events U.S. Department of Health and Human Services, Food and Drug Administration, Center for Biologics Evaluation and Research, November 2006. (3) Guidance for Industry Preclinical Assessment of Investigational Cellular and Gene Therapy Products. Department of Health and Human Services, Food and Drug Administration, Center for Biologics Evaluation and Research, November 2013
2. Describe inclusion/exclusion criteria if samples or animals were excluded from the analysis. Were the criteria pre-established?	Mice included in this analysis were 10-11 weeks of age at dosing; males weighed 20-45gms and females weighed 15-40gms measured within 3 days of arrival. The non human primates were screened for the absence of anti-AAV8 neutralizing antibodies with a cut off titer of <20 to be included in this study. Any animals with any evidence of disease or physical abnormalities were not be selected for study.
3. Were any steps taken to minimize the effects of subjective bias when allocating animals/samples to treatment (e.g. randomization procedure)? If yes, please describe.	Animals (mice) considered suitable for study were randomized into treatment groups using a standard, by weight, measured value randomization procedure. Sample collection from mice was done in an alternating fashion (one animal per sex from each dose group, then repeating) to reduce handling and time biases.
For animal studies, include a statement about randomization even if no randomization was used.	Animals (mice) considered suitable for study were randomized into treatment groups using a standard, by weight, measured value randomization procedure.
4.a. Were any steps taken to minimize the effects of subjective bias during group allocation or/and when assessing results (e.g. blinding of the investigator)? If yes please describe.	For the mouse PAS tissue analysis, the pathologist conducting the microscopic evalution was blinded ot the identity and treatment of the animal represented on the slide. For non human primate and mouse H&E staining analysis, no steps were taken.
4.b. For animal studies, include a statement about blinding even if no blinding was done	Blinding was performed when practical and allowed by experimental design.
5. For every figure, are statistical tests justified as appropriate?	Yes

Do the data meet the assumptions of the tests (e.g., normal distribution)? Describe any methods used to assess it.	N/A
Is there an estimate of variation within each group of data?	N/A
Is the variance similar between the groups that are being statistically compared?	N/A

C- Reagents

6. To show that antibodies were profiled for use in the system under study (assay and species), provide a citation, catalog number and/or clone number, supplementary information or reference to an antibody validation profile. e.g., Antibodypedia (see link list at top right), 1DegreeBio (see link list at top right).	We have provided the requested details on all antibodies used in this study, under the relavant technique in Methods.
7. Identify the source of cell lines and report if they were recently authenticated (e.g., by STR profiling) and tested for mycoplasma contamination.	N/A

* for all hyperlinks, please see the table at the top right of the document

D- Animal Models

8. Report species, strain, gender, age of animals and genetic modification status where applicable. Please detail housing and husbandry conditions and the source of animals.	Mice used in this study were B6;129- Gaa tm1Rabn/J (Gaa-/-) from Jackson Laboratory (Bar Harbor, ME, USA), a Gaa KO strain carrying a targeted deletion of exon 6, and wild-type littermates of the KO mouse model (B6;129). The non human primates (NHPs) were juvenile Chinese Cynomolgus macaques that were received from different vendors and were 2.4 to 3.2 years old and weighed between 2.2 and 2.8 kg on Day -1. Both sexes of animals were used for mouse and non human primate experiments.
9. For experiments involving live vertebrates, include a statement of compliance with ethical regulations and identify the committee(s) approving the experiments.	All protocols involving use of animals were approved by the Institutional Animal Care and Use Committee (IACUC) of Charles River Laboratories at Mattawan, MI (mice) or at Reno, NV (non human primates). All experiments were performed in accordance with relevant guidelines and regulations.
10. We recommend consulting the ARRIVE guidelines (see link list at top right) (PLoS Biol. 8(6), e1000412, 2010) to ensure that other relevant aspects of animal studies are adequately reported. See author guidelines, under 'Reporting Guidelines'. See also: NIH (see link list at top right) and MRC (see link list at top right) recommendations. Please confirm compliance.	Confirmed

E- Human Subjects

11. Identify the committee(s) approving the study protocol.	NA
12. Include a statement confirming that informed consent was obtained from all subjects and that the experiments conformed to the principles set out in the WMA Declaration of Helsinki and the Department of Health and Human Services Belmont Report.	NA
13. For publication of patient photos, include a statement confirming that consent to publish was obtained.	NA
14. Report any restrictions on the availability (and/or on the use) of human data or samples.	NA
15. Report the clinical trial registration number (at ClinicalTrials.gov or equivalent), where applicable.	NA
16. For phase II and III randomized controlled trials, please refer to the CONSORT flow diagram (see link list at top right) and submit the CONSORT checklist (see link list at top right) with your submission. See author guidelines, under 'Reporting Guidelines'. Please confirm you have submitted this list.	NA
17. For tumor marker prognostic studies, we recommend that you follow the REMARK reporting guidelines (see link list at top right). See author guidelines, under 'Reporting Guidelines'. Please confirm you have followed these guidelines.	NA

F- Data Accessibility

18: Provide a "Data Availability" section at the end of the Materials & Methods, listing the accession codes for data generated in this study and deposited in a public database (e.g. RNA-Seq data: Gene Expression Omnibus GSE39462, Proteomics data: PRIDE PXD000208 etc.) Please refer to our author guidelines for 'Data Deposition'.<br><br>Data deposition in a public repository is mandatory for:<br>a. Protein, DNA and RNA sequences<br>b. Macromolecular structures<br>c. Crystallographic data for small molecules<br>d. Functional genomics data<br>e. Proteomics and molecular interactions	A Data Availability section was added to the manuscript. This study includes no data deposited in external repositories.
19. Deposition is strongly recommended for any datasets that are central and integral to the study; please consider the journal's data policy. If no structured public repository exists for a given data type, we encourage the provision of datasets in the manuscript as a Supplementary Document (see author guidelines under 'Expanded View' or in unstructured repositories such as Dryad (see link list at top right) or Figshare (see link list at top right).	N/A
20. Access to human clinical and genomic datasets should be provided with as few restrictions as possible while respecting ethical obligations to the patients and relevant medical and legal issues. If practically possible and compatible with the individual consent agreement used in the study, such data should be deposited in one of the major public access-controlled repositories such as dbGAP (see link list at top right) or EGA (see link list at top right).	N/A
21. Computational models that are central and integral to a study should be shared without restrictions and provided in a machine-readable form. The relevant accession numbers or links should be provided. When possible, standardized format (SBML, CellML) should be used instead of scripts (e.g. MATLAB). Authors are strongly encouraged to follow the MIRIAM guidelines (see link list at top right) and deposit their model in a public database such as Biomodels (see link list at top right) or JWS Online (see link list at top right). If computer source code is provided with the paper, it should be deposited in a public repository or included in supplementary information.	N/A

G- Dual use research of concern

22. Could your study fall under dual use research restrictions? Please check biosecurity documents (see link list at top right) and list of select agents and toxins (APHIS/CDC) (see link list at top right). According to our biosecurity guidelines, provide a statement only if it could.	No, our study does not fall under dual use research restriction.
